# Genotype-Property Patient-Phenotype Relations Suggest that Proteome Exhaustion Can Cause Amyotrophic Lateral Sclerosis

**DOI:** 10.1371/journal.pone.0118649

**Published:** 2015-03-23

**Authors:** Kasper P. Kepp

**Affiliations:** Department of Chemistry, Technical University of Denmark, Kongens Lyngby, Denmark; Universidad de Granada, SPAIN

## Abstract

Late-onset neurodegenerative diseases remain poorly understood as search continues for the perceived pathogenic protein species. Previously, variants in Superoxide Dismutase 1 (SOD1) causing Amyotrophic Lateral Sclerosis (ALS) were found to destabilize and reduce net charge, suggesting a pathogenic aggregation mechanism. This paper reports analysis of compiled patient data and experimental and computed protein properties for variants of human SOD1, a major risk factor of ALS. Both stability and reduced net charge correlate significantly with disease, with larger significance than previously observed. Using two independent methods and two data sets, a probability < 3% (t-statistical test) is found that ALS-causing mutations share average stability with all possible 2907 SOD1 mutations. Most importantly, un-weighted patient survival times correlate strongly with the misfolded/unfolded protein copy number, expressed as an exponential function of the experimental stabilities (*R* ^2^ = 0.31, *p* = 0.002), and this phenotype is further aggravated by charge (*R* ^2^ = 0.51, *p* = 1.8 x 10−5). This finding suggests that disease relates to the copy number of misfolded proteins. Exhaustion of motor neurons due to expensive protein turnover of misfolded protein copies is consistent with the data but can further explain e.g. the expression-dependence of SOD1 pathogenicity, the lack of identification of a molecular toxic mode, elevated SOD1 mRNA levels in sporadic ALS, bioenergetic effects and increased resting energy expenditure in ALS patients, genetic risk factors affecting RNA metabolism, and recent findings that a SOD1 mutant becomes toxic when proteasome activity is recovered after washout of a proteasome inhibitor. Proteome exhaustion is also consistent with energy-producing mitochondria accumulating at the neuromuscular junctions where ALS often initiates. If true, this exhaustion mechanism implies a complete change of focus in treatment of ALS towards actively nursing the energy state and protein turnover of the motor neurons.

## Introduction

An urgent challenge in biology and medicine is to translate the vast amount of genomic data now available into phenotypes, preferably by mapping genetic variations via the transcribed protein properties to organism-level phenotypes. Such a mapping could substantially accelerate the understanding of disease mechanisms, prospects of early diagnosis, and personalized treatments reflecting the specific genotypes of individual patients, and could enable a new era of “smart” disease management.

Amyotrophic Lateral Sclerosis (ALS) is the most common degenerative disease of the motor neurons: It initiates within limbs or bulbar muscles and is eventually lethal due to collapse of muscular breathing function[1][2][3][4]. As other complex late-onset neurological disorders, no effective treatment exists and current drugs delay disease progression by only some months[1]. ALS manifests in two forms: Familiar ALS (FALS) with inherited risk genotypes accounts for ∼10% of cases and sporadic ALS (SALS) without apparent heritability accounts for ∼90% of cases[1][3][5]. Whereas the median age of ALS diagnosis is mid-to-late fifties[1][6], it is commonly a decade earlier for FALS[3], although very dependent on the variant.

Neurological disorders share molecular pathological features such as deposition of protein aggregates, metal dyshomeostasis, mitochondrial and oxidative stress, inflammation, and apoptosis[7][8][9] and the aging phenotype is central to disease manifestation, which is aggravated by multiple genetic, life-style, and environmental risk factors[9][10]. Thus, a spectrum of phenotypes exist even in families sharing the same genetic risk variant.

Genetic risk factors constitute the primary framework for understanding disease mechanisms[5]. Up to 2/3 of the FALS cases correlate with variations in ∼15 genes[1][5], the two most important being Superoxide Dismutase 1 (SOD1, explaining 10−15% of FALS cases[1][3][5][11]) and the hexanucleotide repeat expansion in open reading frame 72 on chromosome 9 (C9ORF72), explaining up to 40% of FALS cases in Europe and North America[12]. The first was identified twenty years ago[13] and the latter only identified in 2011[14][15]. SALS also has some (∼10%) genetic background[5][16]. Both SOD1 and C9ORF72 have roles also in SALS[12,14], and recently identified genetic risk factors point to roles of RNA metabolism (TAR-DNA binding protein 43[17], FUS[18][19]) and protein processing (SQSTM1[20], VCP[21]) in the disease[5].

**Fig 1:**
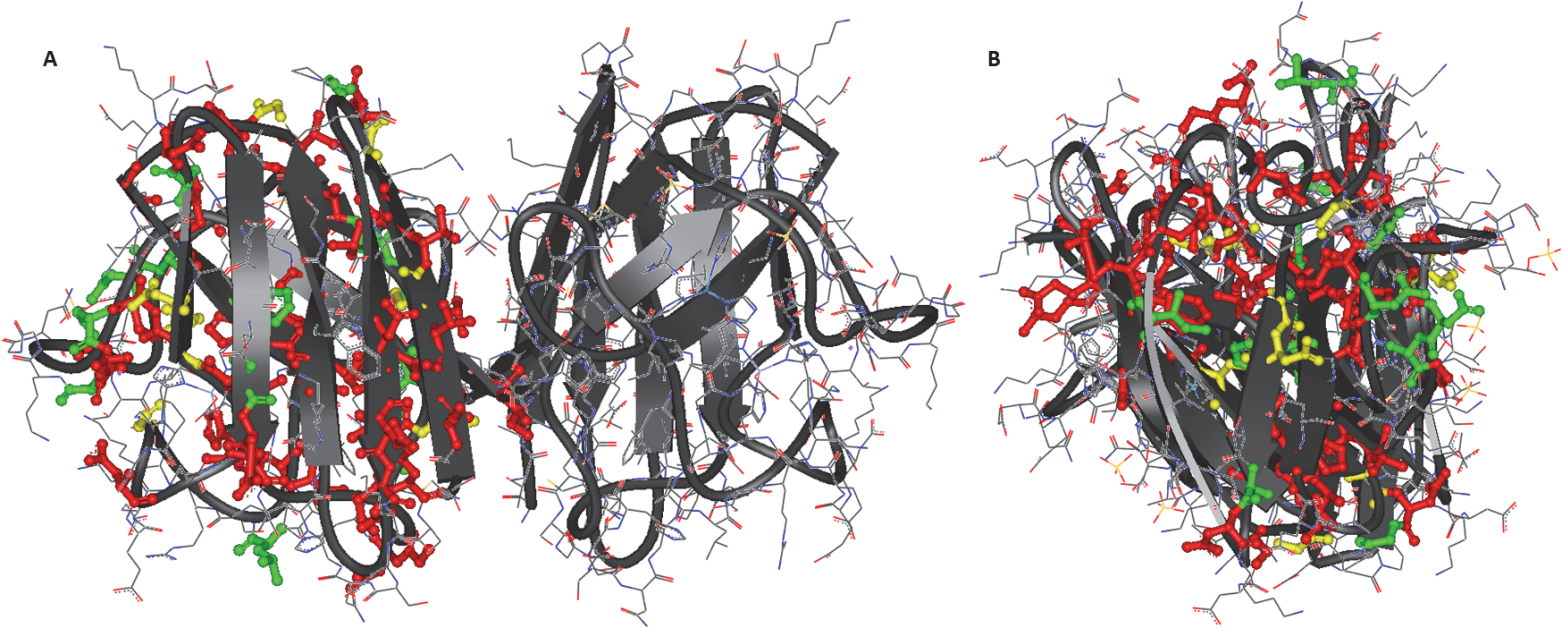
64 Sites in human SOD1 subject to 77 missense variants for which patient age of ALS onset t(o) has been reported, out of 150 variants studied in this work. **A)** seen from the dimer longside perspective. **B)** seen from the end perspective. Sites are marked in ball-and-stick on the protein structure (2C9V.pdb[26]) according to t(o) < 55 (red), t(o) > 55 (green), or sites with variants showing phenotypes with both t(o) > 55 and t(o) < 55. See Table I in S1 File for details. Figure made with ViewerLite 4.2, Accelrys.

As a systemically important, highly expressed and stable protein, the knowledge of SOD1 is substantial, making it a central framework for understanding ALS[11]. Also, the severity of some SOD1-variants such as A4V suggests that SOD1-variants are likely to reveal pathogenic insight. The protein, shown in Fig. 1, is one of three human SOD isoforms that protect against oxidative stress caused both by exposure and the mitochondria's normal secondary production of O_2_^-^ [22][23]. SOD1 is a homo-dimer consisting of two β-barrel (Greek key) monomers with Cu and Zn in the active site. SOD1 performs two half-reactions in a catalytic cycle that oxidizes two toxic superoxide radicals O_2_^-^ to one molecule of O_2_ and one H_2_O_2_, while the catalytic metal ion Cu shifts between oxidations states I and II[22][24].

The variants causing FALS are found across the protein[22][25]: Fig. 1 shows all sites representing variants with known ALS patient data, as collected in this work (only shown for one subunit for clarity) mapped on the high-resolution structure 2C9V.pdb[26]: Red colors represent age of onset < 55 years, green > 55 years, and yellow mixed phenotypes. As seen, the sites and colors spread across the entire protein, i.e. there is no clear relationship between pathogenicity and structural location (full data in S1 File). The commonly cited 2−3[5] years of survival time covers a spread from less than a year to ∼20 years (see data later in this paper), providing enlightening variability in phenotypes that facilitate genotype-phenotype analysis.

Mice without wild-type SOD1 do not normally develop ALS[1], and FALS is autosomal dominant[11], i.e. presence of wild-type SOD1 in heterozygotes does not prevent FALS as seen from co-expression[27]. Thus, SOD-1 mutants are considered to gain an unknown toxic function[1][3][22] relating to e.g. redox toxicity or toxic aggregation, although the specific toxic mechanism and species have not been identified[28][29][30]. Several reports found that instability of SOD1 variants correlates with disease severity[31][32][33] and many SOD1 mutations do reduce stability[22][34][35], consistent with pathogenic aggregation. However, average instability is expected, as a typical mutation destabilizes by ∼1 kcal/mol[36]. Some ALS-causing SOD1 mutations do not reduce stability of the aggregation-prone apostates, but may still increase aggregation propensity due to e.g. changes in hydrophobicity or net charge[29][33][37], although data from 13 ALS-causing variants correlated poorly with disease duration[38]. Aggregation propensity was found to relate to disease duration but not significantly (*p* = 0.14, *R* ^*2*^ ∼0.23) and not with age of onset[37]. Misfolding is coupled to metal release[25][39], and mutations may reduce metal content[40][41] to destabilize the holoprotein but not the apoprotein. Still, experimental holodimer and apomonomer stabilities are highly correlated (*R* = 0.83)[42], so state-specific effects constitute only part of the picture, importantly undermining the idea of one particular (i.e. state-specific) toxic protein species.

In this work, the latest reported patient data and experimental and computed biochemical properties of SOD1 variants have been collected and analyzed for 150 missense mutations, including 77 variants with patient data and 30 with experimentally known stabilities. Using the expanded data set, stability and charge correlate strongly with disease phenotype, even more strongly than previously found[33]. Second, by using a new approach that computes directly the stability of all possible mutations in the protein and compares these with the stabilities of the disease-related mutants, a new statistical test is provided that shows that stability is a significant factor at the 97% confidence level. Third, it is shown that patient phenotypes correlate more strongly with the copy number of misfolded proteins derived directly from experimental stabilities without parameterization than with stability itself as previously investigated, providing the strongest correlation so far identified from un-weighted data. Based on this, a new mechanism of neurodegeneration resulting from general exhaustion of motor neurons is suggested that can reconcile a range of observations and provide a completely new framework for researching and treating ALS and possibly other neurodegenerative diseases.

## Methods

### Collection of data

Experimental free energies of folding (ΔΔG) were collected from Vassal et al.[38], Nordlund et al.[43], Lindberg et al.[32], Stathopulos et al.[44], and Byström et al.[29] SOD1 variants causing ALS and their associated patient data were collected from the compiled data from the ALS online genetics database[45] and from Wang et al.[33] after removing redundant data. Additional phenotype data were collected for recently studied variants such as K3E[46], V31A[47], R115C[48], C7W[49] and several others: Table I of the S1 File shows the compiled stabilities and patient data of SOD1 variants with references. In total, 150 single-site missense mutations (non-synonymous substitutions) were compiled, of which 77 variants have associated patient data, providing the most complete ALS-SOD1 data base yet studied.

### Nature and heterogeneity of collected data

Heterogeneity of phenotype data is a major issue in genotype-phenotype correlations for late-onset multifactor diseases such as neurological diseases, because the risk modifiers from other genes, life style, and environment increase data noise[9]. Also, to put the data into clinical context, one should consider the frequencies of each variant and its overall vs. regional contribution to disease.

Regional dependence demonstrates the role of additional risk modifiers: The frequency of variants is highly region-dependent, with the large frequencies of A4V seen primarily in the United States. In recent analysis, 92 out of 1220 ALS cases in the United States had SOD1 mutations (ALSSOD)[50]. Of these, 39 (42%) were A4V and 9 (10%) were I113T. Review of patient data from Alberta, Canada suggested that I113T is also common in this area, with 11 independent observations out of 47 SOD1 variants identified, whereas A4V did not show up[51], revealing substantial regional dependence.

Also, major heterogeneity in reported patient phenotypes exists (Table I of S1 File). Despite the heterogeneity and large standard deviations, significant genotype-phenotype correlations were previously identified after weighting data according to the number of patient observations *n*[33]. There are various ways to reduce noise in a data: The statistically simple one is to de-emphasize data with too large spread or to small *n*, since a few observations are most likely insignificant, as seen e.g. from the heterogeneous data reported from families of only two affected members[52]. The previously used approach of weighting data by *n* partly solves this problem[33]. However, due to the very large spread in *n*, such weighting reduces correlation to effectively only the very most abundant variants such as A4V, H46R, E100G, and I113T (*n* > 50, see Table I in S1 File) at a drastic loss of information for the many low *n* phenotypes.

### Analysis of data

Patient data for both age of onset, t(o), and survival times, t(s), were analyzed, together with age of death, t(d) = t(o) + t(s). t(o) is affected by uncertainties in time of diagnosis (i.e. when did symptoms actually begin). Also, some variants reflect late-onset but rapid disease progression, which some researchers might characterize as severe, others as mild. However, as age increases, the general fitness of the patient will play a role, and risk modifiers will play out strongly in the total phenotype. Thus in principle, a survival time t(s) of 2 years after an onset t(o) at 60 years is not as severe as it would be if the patient had t(o) = 50 years. In contrast, early-onset, long-duration phenotypes could be classified as severe if only t(o) was used, even if the duration postpones death beyond other late-onset variants. Thus, it is of interest to also study a third phenotype, t(d) = t(o) + t(s), as this measure potentially resolves some of these complications.

These patient data were correlated against collected experimental stabilities, computed stabilities (see below), and the following additional computed properties: Changes in net charge, hydrophobicity, beta and alpha propensities and logarithms thereof (to convert into free energy scale), and any of these weighted by their solvent accessible surface of the mutated site, since any pathogenic property might depend on its solvent exposure. Correlations were carried out linearly and with logarithms, and subjected to regression analysis. Statistically significant correlations were reported with correlation coefficients and *p*-values.

### Computing the misfolded copy numbers resulting from SOD1 variants

Due to the strong correlations found between patient data and SOD1 stability and charge, and the implied absence of simple state-specific toxicities, an alternative systemic pathogenic model was investigated. We have recently described a proteostatic maintenance model that can explain a range of trends in properties of proteomes suggesting selection to minimize proteostatic costs, since energy spent on the proteome makes up a large fraction of the total energy budget of cells[53]. The proteostasis of protein *i* can be described by the simple kinetic model[53]:

$$mRNA_{i}\overset{k_{s_{i}}}{\to }F_{i}\overset{k_{1_{i}}}{\underset{k_{2_{i}}}{⇌}}U_{i}\overset{k_{d_{i}}}{\to }D_{i}$$ (1)

Here, *F*_*i*_ is folded protein copies, *U*_*i*_ is unfolded copies, and *D*_*i*_ are degraded peptide fragments, with respective kinetic constants. Since *k_s_i__* is constant, it requires higher expression rate per time unit. For an abundant protein, this cost may exhaust motor neurons already stretched by other energy demands. The cellular maintenance energy (in J s^-1^) allocated to one protein *i* per time unit can be estimated using the equation by Kepp and Dasmeh[53] (please notice that the original equation has a factor 2 error that does not affect the model):

$$dE_{m}∕dt=A_{i}\left(\frac{1}{1+\exp\left(\frac{-\Delta G_{i}}{RT}\right)}\right)k_{d_{i}}N_{aa_{i}}\left(C_{s_{i}}+C_{d_{i}}\right)$$ (2)

This equation also defines proteostatic exhaustion as an increase in the energy required to maintain proteostasis, primarily caused by increased turnover costs. In this equation, *A*_*i*_ is the total protein abundance, Δ*G*_*i*_ is the stability, and *N_aa_i__, C_s_i__*, and *C_d_i__* are the number of amino acids in protein *i* and the average synthetic and degradation cost (in units of J) per amino acid in protein *i*[53]. The four parameters *K_d_i__N_aa_i__*(*C_s_i__* + *C_d_i__*) were simplied as one constant, *c*_i_ = 10^-7^, a reasonable value derived for protein turnover. This maintenance cost can be shown to act directly on the amount of misfolded protein, since $U_{i}=A_{i}\left(\frac{1}{1+\exp\left(\frac{{-∆G}_{i}}{RT}\right)}\right)$. Any change in energy costs is therefore proportional to Δ*U*_i_, which can be computed form experimental stabilities of the variants. This expression was used to compute *dE*_m_/*dt* for both wild-type and SOD1 variants. Realistic values of *A*_i_ and ∆*G*_i_/RT were chosen to be 100,000 and −25. Importantly, these constants do not affect the statistical correlation which depends only on the relative change in cost: This phenotype is a function of the stability, and thus, contains no adjustable parameters and the same accuracy as the experimental stabilities.

### Calculation of variant stabilities

To enable a statistically significant assessment of the role of protein stability and charge in ALS, these properties were computed for all the 150 SOD1 variants including the 120 where experimental stabilities are not available. For this purpose, two methods were used, POPMUSIC 2.1 [54][55] and I-MUTANT 2.0 [56][57], which provide accurate descriptions of the stabilities of SOD1 mutants, compared to several other methods[42]. The structure used for calculation was the high-resolution structure 2C9V.pdb[26], which produced accurate stabilities in recent work[42]. Importantly, although local variations will occur when using distinct crystal structure templates, the results obtained with these two methods are generally structure-insensitive compared to other stability calculators[42].

Human protein variants represent arising mutants that on average have certain characteristics such as destabilizing tendency, since they mostly impair the fitness-optimal properties of the wild type. This bias will affect genotype-phenotype correlations and should thus be considered by comparing disease-causing variant properties against not just the wild type but also the full background of all possible mutations. With the advent of fast protein property calculators, such a procedure is now feasible. This work explains how they can be used to substantially increase the significance of genotype-phenotype correlations due to i) large numbers compared to experimental data, and ii) cancellation of systematic errors in computation when comparing variant sets. In SOD1, the full background set amounts to 2907 mutations (19 x 153) that were previously computed with POPMUSIC for matters of calibrating computational methods[42]. When comparing the distributions of property changes of the disease variants against all possible variants, a student’s t-test can be performed on the distributions to investigate the null hypothesis that the distribution means are identical. This type of analysis provides a useful tool for genotype-phenotype correlation analysis that compliments linear regression analysis.

## Results and Discussion

### General trends in patient data of SOD1 variant carriers

Table I in S1 File shows the compiled data for 150 missense substitutions in SOD1. For each mutation, the classification of the site as beta sheet, metal-binding region, or cysteine bridge region is given, together with the solvent-accessibility of the mutated site calculated by POPMUSIC2.1 (using the high-resolution structure 2C9V.pdb[26]), the experimental monomer or dimer free energy changes relative to the wild type, if available, the number of patients *n* from whom the phenotype is estimated, and the phenotypes of age of onset t(o), survival time t(s), and age of death t(d), all in years.

The total number of patients *n* is 1053. However, due to the large variations in frequencies of the variants, some variants contribute highly to these data, with four variants having *n* > 50: A4V (212), H46R (70), E100G (54), and I113T (53). The statistical significance of the phenotype estimates is very dependent on *n*, but also depends on the severity of the variant, with more severe variants having less variable disease durations, as other risk factors play a reduced role in these cases. Also, in some reports, only t(o) is reported since patients have not been monitored until death. These 36 onset ages are not analyzed further in this work but are reported in S1 File for completion.

When weighting each variant’s phenotypes with their observed frequency share of the remaining 1017 observations, one obtains the following averages for SOD1-associated ALS: t(o) ∼ 47.5 years, t(s) ∼ 5.8 years, and t(d) ∼ 53.3 years. If one considers the variant average without weighting by the frequency of each variant, the numbers would be quite similar: t(o) ∼47.4 years, t(s) ∼5.5 years, and t(d) ∼53.6 years. These data can be compared with the SALS figures of t(o) ∼ 58 years, showing that on average, SOD1 variants reduce t(o) by roughly 10 years.

### Changes in SOD1 stability and net charge correlate with ALS patient survival times

**Fig 2:**
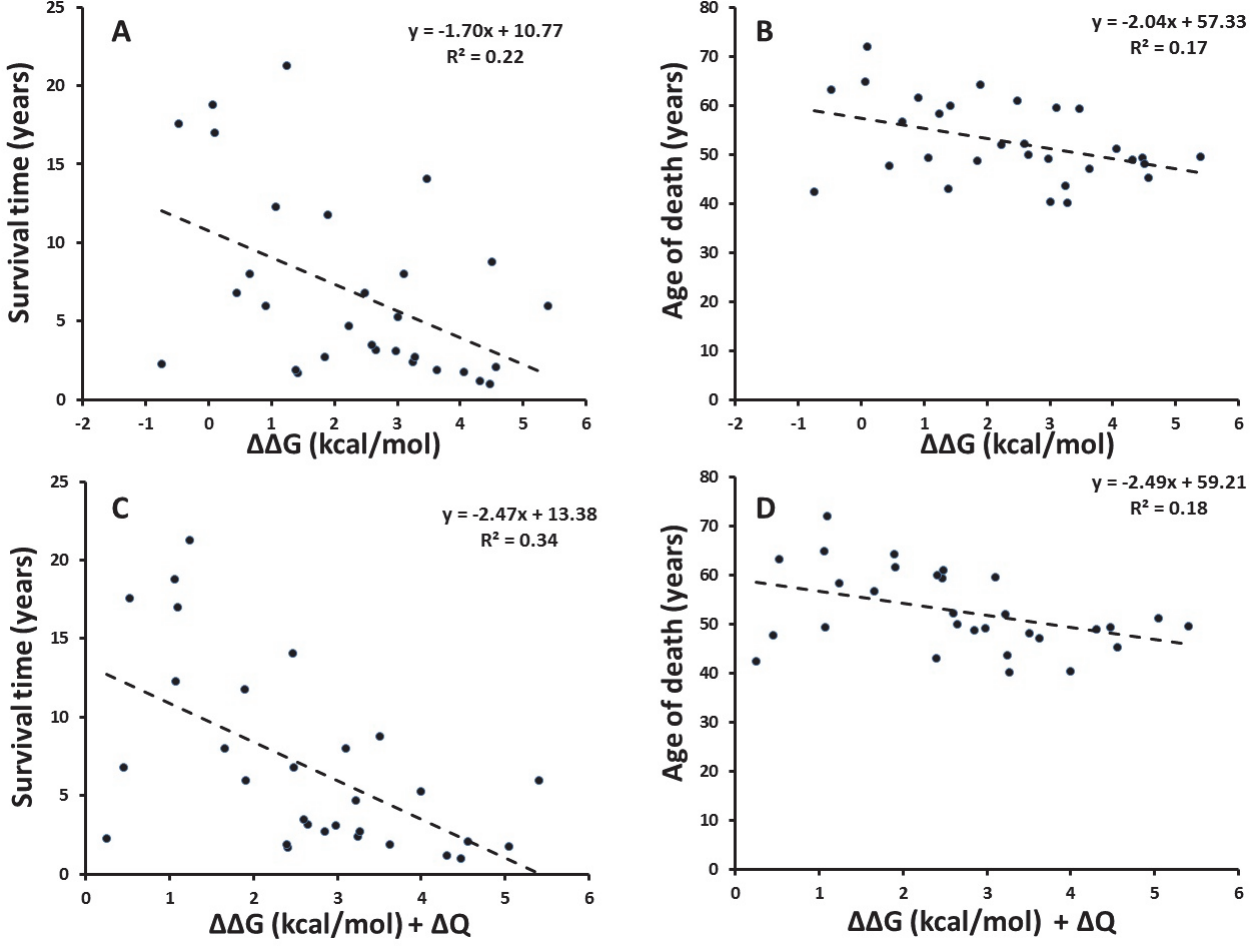
Patient survival time and age of death vs. stability and charge changes. Correlation between (**A**) average survival time or (**B**) age of death (in years) of ALS patients carrying any of 30 SOD1 variants vs. the corresponding experimental stability changes (kcal/mol). (**C**) average survival time and (**D**) age of death correlated against combined stability and net charge changes.

Fig. 2 shows the correlation between disease phenotypes t(s) and t(d) and experimental stability changes (ΔΔG) of holodimers with or without account for charge variation, for the 30 variants where experimental stabilities are available (numerical data are given in S1 File). The comparison for t(s) in Fig. 2A is an updated version of the analysis previously done by Wang et al. using 28 data points[33], now augmented with additional patient data.

Notably, disease onset t(o) did not correlate with stability changes, as also found previously[33]. However, as previously reported, disease duration t(s) correlates significantly with the experimentally measured stability change of the dimer SOD1 (*R* ^2^ ∼ 0.22, *p* ∼ 0.0081, standard error ∼5.2 years, Fig. 2A). With the new data available used, this correlation is considerably strengthened compared to the previously reported *R* ^2^ ∼0.12 (*R* = 0.34) for un-weighted data (the correlation increases if data are weighted by *n*, as mentioned in the Methods section[33]), and now the correlation is significant at the 95% confidence level, which it was not previously. Furthermore, also the age of death t(d) = t(o) + t(s) correlates significantly with stability increases (*R* ^2^ ∼ 0.17, *p* ∼ 0.024, standard error 7.5 years, Fig. 2B). Thus, the present analysis reveals the first statistically significant correlations at the 95% confidence level for un-weighted ALS patient data, showing clearly the importance of protein stability in SOD1 pathogenicity.

Furthermore, charge has previously been found to be an important co-determinant of pathogenicity of SOD1 variants[29][33][37]. To appreciate this, correlating the sum of ΔΔG and the change in net absolute charge │ΔQ│of the SOD1 variant markedly improved correlation vs. t(s) without any parameterization (*R* ^2^ ∼ 0.34, *R* = 0.58, *p* ∼ 0.00080, standard error ∼4.9 years, Fig. 2C) with t(d) correlations being similar (Fig. 2D). If this two-property linear fit is optimized (ΔΔG + 1.7ΔQ), *R* ^2^ increases to 0.37. Thus, this analysis, using updated patient data without any weighting substantially strengthens previous conclusions that both protein stability and net absolute charge play major pathogenic roles in SOD1-associated ALS. Whereas a positive ΔΔG increases the tendency of the protein to misfold and unfold, reductions in net negative charge of SOD1 are likely to increase aggregation propensity, as evident e.g. from the work by Chiti and Dobson et al.[58]

A final observation from the analysis summarized in Fig. 2 is that the mild phenotypes are responsible for most of the outliers, with a triangular shape of outliers observed in all four regression plots. This documents a “mild-phenotype” noise effect that should be considered in future studies of late-onset diseases such as neurological diseases. The most likely explanation for this effect is that mild phenotypes arise from genetic variants with a smaller contribution to the total risk (which, in general, is a sum of genetic, environmental, and life-style risk factors), thereby increasing the noise from other risk modifiers, both genetic and non-genetic.

### Significant destabilization in known SOD1 variants vs. the mutation background

The limited number of mutants with experimentally measured stabilities prevent a full-scale investigation of the role of stability across known SOD1 variants. Furthermore, mutations are on average likely to be destabilizing by at least 1 kcal/mol[36], so it is relevant to investigate the stability of ALS-causing SOD1 variants in the context of the “expected” stability change of a random SOD1 mutation.

To obtain such an estimate, we used two methods, POPMUSIC 2.1 and I-MUTANT 2.0, recently shown to provide the most accurate descriptions of stabilities of SOD1 mutants with experimentally available stabilities [42]. For the mutations studied in this work, the correlation coefficient between POPMUSIC and experiment is 0.52 (*R* ^2^ = 0.27). Furthermore this increases to 0.70 (*R* ^2^ = 0.49) if only one outlier is removed (the A4V variant with the large experimental destabilization energy of 4.3 kcal/mol; see S1 File).

While computational methods are generally subject to substantial errors[42], comparison of relatively large data sets computed by the same method will reduce systematic errors and, using the correct physics that is present in the models, provide a strong tool for estimating the significance of a potentially pathogenic property against the total mutation background. This mutation-background test has not been applied before and is a valuable compliment to linear regressions. The MAEs of POPMUSIC and I-MUTANT are approximately 1 kcal/mol [42] and smaller when the systematic error is removed by comparison of the two variant sets. Second, errors in individual site calculations will be less important because data for all variants can be calculated. One can then use Student’s t-test for comparing the two distributions, providing a further test of pathogenic hypotheses.

Similarly, ΔQ for all possible mutations was computed from the possible changes based on existing charges of amino acids (e.g. 30 mutations would reduce charge by two, corresponding to D or E substitutions of one of the 15 positively charged sites. There are 117 neutral sites, giving 117*15 = 1755 mutations with ΔQ = 0 from these, + 15 and 21 from the positive and negative sites. In total, 1791 mutations do not change charge, 474 decrease it by 1, 570 increase it by 1, 30 decrease by 2, and 42 increase by 2). These values of ΔQ provide a background distribution for comparison to the ΔQ values observed for ALS-related mutations.

**Fig 3:**
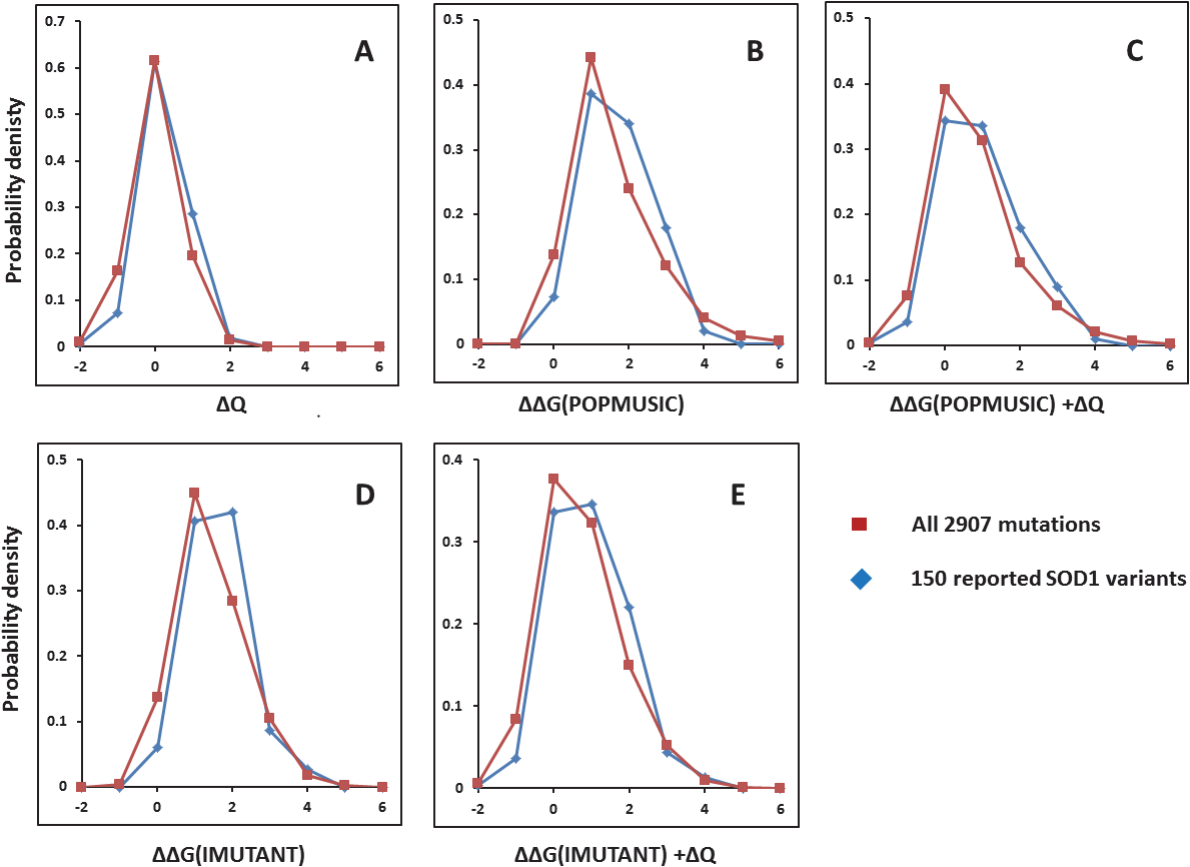
Putative ALS mutations vs. all possible mutations. Distributions of all 150 known missense SOD1 mutations relating to ALS (blue) vs. background distributions of all 2907 possible missense mutations (red). (**A**) charge change from sequence (ΔQ); (**B**) free energy change (ΔΔG) computed with POPMUSIC; (**C**) ΔΔG computed with IMUTANT; (**D**) free energy (IMUTANT) plus charge change; (**E**) free energy (POPMUSIC) plus charge change.

Fig. 3 shows the result of comparing the change in charge (ΔQ), stability (ΔΔG), or both, for all 150 variants (blue) against the background distribution of all possible 2907 SOD1 mutations (red). As seen, the ΔQ distributions are similar with a small tendency towards reduced net charge in the reported SOD1-variants. In contrast, ΔΔG computed with both POPMUSIC (Fig. 3B) and I-MUTANT (Fig. 3D) differ significantly from expectation and are shifted towards less stability. When ΔQ and ΔΔG are considered together, this picture prevails (Fig. 3C and 3E). Since this result is obtained with two distinct methods, it further documents the robustness of the mutation-background test. From POPMUSIC, the average stability of the 150 SOD1-variants is 1.21 kcal/mol vs. 1.05 kcal/mol for all possible mutations. From I-MUTANT, these numbers are 1.10 kcal/mol and 0.91 kcal/mol, showing the same tendency. With both methods, the stabilities of reported SOD1 variants differ significantly at the 95% confidence level from the expected background stability (t-test, two-tailed, non-equal variances). These data are compiled in Table 1.

**Table 1:** Statistics after test for same mean of ALS-causing SOD1 mutations and all 2907 possible mutations (student’s t-test, two-tailed, different variances).

	POPMUSIC 2.1		I-MUTANT 2.0	
	*All reported 150 mutations*	*All possible 2907 mutations*	*All reported 150 mutations*	*All possible 2907 mutations*
avr ΔΔG (kcal/mol)	1.21	1.05	1.10	0.91
variance (kcal/mol)	0.78	1.15	0.60	0.81
*p*-value	0.030	—-	0.0051	—-
	*60 mutations with n ≥ 3*	—-	*60 mutations with n ≥ 3*	—-
avr ΔΔG (kcal/mol)	1.32	—-	1.18	—-
variance (kcal/mol)	0.74	—-	0.52	—-
*p*-value	0.021	—-	0.0058	—-

**Fig 4:**
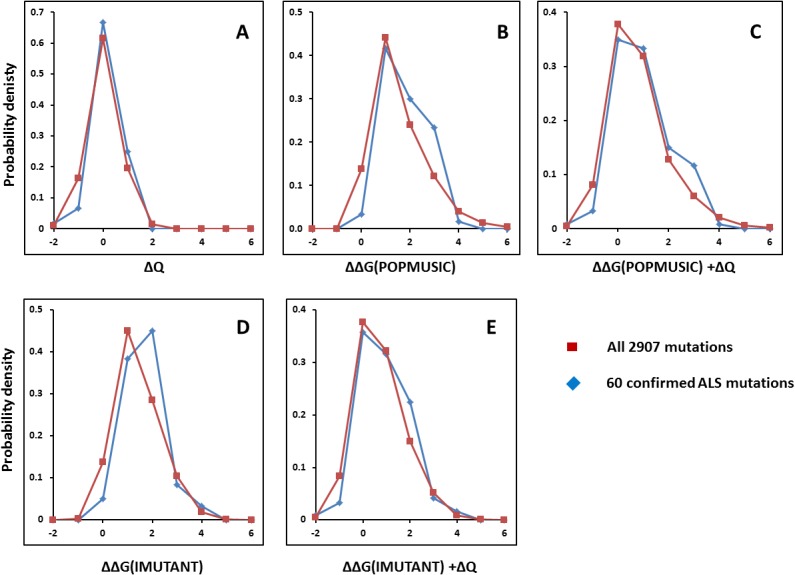
Confirmed ALS mutations vs. all possible mutations. Distributions as in Fig. 3, but based on the 60 SOD1 mutations with known patient data relating to ALS (blue) vs. background distributions of all 2907 possible missense mutations (red). (**A**) charge change from sequence (ΔQ); (**B**) free energy change (ΔΔG) computed with POPMUSIC; (**C**) ΔΔG computed with IMUTANT; (**D**) free energy (IMUTANT) plus charge change; (**E**) free energy (POPMUSIC) plus charge change.

Since the conclusion drawn above could depend on the choice of the 150 variants, the same analysis was performed with the 60 variants for which ALS clinical phenotypes have been confirmed for at least 3 persons (n ≥ 3). The results, shown in Fig. 4, are fully consistent with the general picture found for all 150 variants, with the notable difference that the 60 documented ALS-causing variants show a further reduction in stability of 0.08−0.11 kcal/mol relative to the mutation background, having average ΔΔGs of 1.32 and 1.18 kcal/mol according to POPMUSIC and I-MUTANT, respectively. These findings are highly significant (*p* = 0.021 and 0.0058 for the POPMUSIC and I-MUTANT analysis, respectively).

Thus, even while mutations generally tend to destabilize, the observed stabilities of reported SOD1 variants are significantly lower than the 2907-mutation background of SOD1, and this effect is emphasized further in the confirmed ALS-causing variants where patient data are available. In conclusion, by using two different statistical tests, one for linear regression and one for comparison of distributions, it is shown that reduced stability and net charge correlate with ALS pathogenicity at the 95% confidence level, the strongest evidence so far reported for their implication in ALS.

### Systemic and non-specific causes of ALS: The role of protein expression

As described above, using compiled patient data and both experimental and computed protein properties, this work establishes protein stability and charge as major pathogenic properties of ALS, with statistical support from two different analyses at the 95% confidence level without any weighting of raw data. As evident from the location of ALS-causing mutations (viz. Fig. 1), the structural context is relatively unimportant, as also implied by the high correlation of dimer and monomer experimental stability data [42]. Thus, specific local modes of molecular action are not likely to cause disease. Instead, the global properties of protein stability and aggregation tendency (as implied by net protein charge) explain patient data. Furthermore, the late-onset multi-risk nature of ALS points to some systemic mechanism at play that has previously been overlooked. The question then naturally arises: How does protein destabilization or aggregation become pathogenic if not by a structurally distinct toxic molecular mechanism?

SOD1 is one of the most abundant proteins in humans, and particularly expressed in energy-requiring cells such as neurons and motor neurons that have high mitochondrial respiration levels and thus, high production of superoxide [22][23]. Given this systemic role of SOD1, other disease mechanisms than commonly discussed toxic molecular species *per se* might be envisioned.

Some hints to a systemic disease mechanism come from the apparent inconsistencies in SOD1 expression data: Higher SOD1 activity has been observed in transgenic mouse models due to overexpression, whereas many SOD1 variants in fact have reduced specific activity[59]. A central, but apparently overlooked observation from multiple studies is that at *high* expression levels, co-expression of wild-type-SOD1 with A4V[60], G85R[61], and G93A[62] variants in fact *aggravate* pathogenicity. However, at *lower* expression levels, in the study by Bruijn et al.[27] similar mouse survival was measured for G85R carriers on a normal wild-type-SOD1 background and on a knockout- wild-type background, i.e. wild-type-SOD1 did not affect the G85R phenotype significantly. These data are consistent with gain of toxic function but also clearly show expression-dependence and that wild-type and mutant both aggravate toxicity at high expression levels: These observations are currently unexplained.

### Proteostatic exhaustion from misfolded SOD1 turnover can explain ALS patient data

The apparently contradictory observations requires a mechanism that, in the limit of high expression levels, has protein copy number as an important pathogenic property. The steady-state copy numbers of misfolded and folded proteins can be estimated directly from cell-specific and protein-specific parameters, and more importantly, the cellular energy cost of handling a protein scales linearly with the total copy number of misfolded proteins[53]. Thus, protein overexpression rather than any specific molecular toxicity is a plausible cause of ALS: If SOD1 is overexpressed and destabilized, higher turnover of degradation-prone proteins variants and higher steady-state copy numbers will increase proteostatic maintenance costs[53].

**Fig 5:**
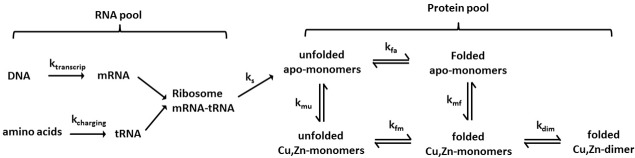
Kinetic scheme describing processes involved in SOD1 proteostasis. Both the RNA pool and the protein pool contribute to proteostatic costs and can thus both involve risk factors in a proteostatic exhaustion mechanism.

From this mechanism, the pathogenicity of a protein variant depends on its combined proteostatic burden, which depends on its degradation and synthesis costs, its turnover rate, and notably, on the amount of misfolded protein which can be estimated from the thermodynamic stability. This energetic burden is expressed in a simple form by Equation (2). The simple turnover Equations (1) constitute a minimal framework required to explain these costs. The more general situation is given in Fig. 5, which accounts for the most important processes of SOD1 turnover. This larger scheme is required if one is to understand the mechanistic context of all the genetic risk factors associated with the neurological disease, as some genetic risk factors affect the RNA turnover and others the protein pool.

When using Equation (2) directly as a framework for understanding neurodegeneration, reduced protein stability as well as expression levels directly increase *dE*_*m*_/*dt*. Equation (2) takes the form of an energy cost that scales linearly with the number of misfolded proteins *U*_i_, and at steady state, this number can be derived from the stability, if other variant properties do not change significantly. The relative cost of a protein variant can be directly studied by this model by comparing *dE*_*m*_/*dt* of variant and wild type, to estimate the increase in proteostatic cost associated with the variant[53].

To estimate the role of proteostatic energy in ALS, Equation (2) was used to compute The misfolded copy numbers *U*_i_ before and after mutation at steady state, and from these, *dE*_*m*_/*dt* for each SOD1 variant for which experimental ΔΔG is available, by using *A*_i_ = 100,000 and the wild type stability of ΔG/RT = −25 (See Table IV in S1 File for numerical data). Importantly, these numbers do not affect the correlation statistics, which only depend on the relative values of *U*_i_ from the exponential function of ΔΔG multiplied by the constants in Equation (2). As seen from Table IV in S1 File, for the more destabilizing variants, the misfolded copy numbers increase by more than 1000-fold. This calculation reveals the burden of misfolded proteins inside a typical cell expressing a highly destabilizing SOD1 variant even if total copy numbers *A*_i_ are unaffected. From these changes in *U*_i_ associated with a variant, the energy cost is estimated from Equation (2) using a conversion constant *c*_i_ = 10^-7^, which is a realistic typical value derived previously from cell-specific turnover data such as life time, synthetic cost, and degradation cost[53].

**Fig 6:**
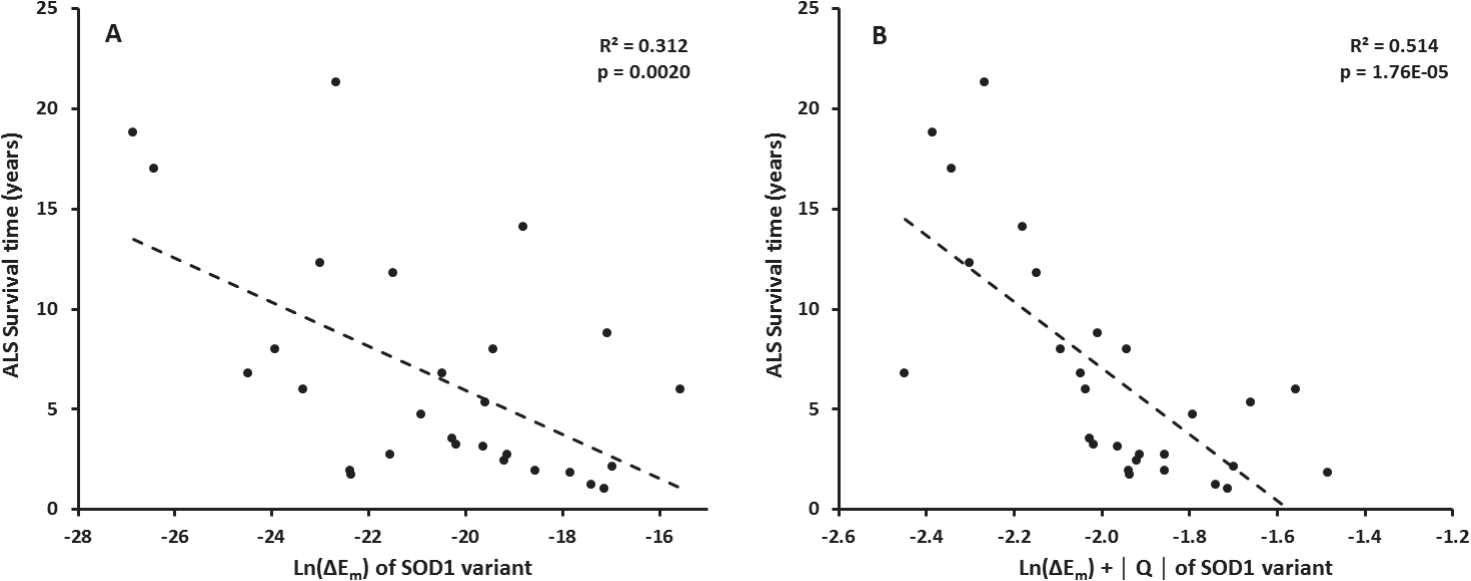
Correlation of survival times t(s) of ALS patients vs. the change in proteostatic energy cost associated with SOD1 variants from Equation (2). (**A**) without and (**B**) with charge changes included.

The results of these calculations excluding or including charge are shown in Fig. 6. It can be seen that mild phenotypes remain outliers in the data (notably the variant I104F with a reported t(s) of 21.3 years), which is expected. However, converting stabilities into misfolded protein copies via Equation (2) reveals a substantially stronger and highly significant correlation (*R* ^2^ = 0.31, *p* = 0.002) to patient survival times than stability alone. If charge is included in the regression, this correlation is substantially further improved, providing the strongest two-property correlation to un-weighted patient data so far observed (*R* ^2^ = 0.51, *p* = 1.8 x 10^-5^).

This analysis shows that protein misfolding diseases such as ALS are not necessarily caused by specific molecular toxicity of misfolded protein species, but possibly by systemic exhaustion due to elevated protein turnover. This mechanism could explain why identification of such malicious protein states has so far been unsuccessful[1][4][9].

### Recent findings are explained by the exhaustion mechanism of neuro-degeneration

Many recent discoveries are consistent or can be directly explained by the exhaustion mechanism proposed in this paper.

1. First, it has been established that ALS patients early in their disease histories experience significantly increased resting energy expenditure that is currently not understood[63]^,^[64]. This finding is directly explained by the proposed mechanism, where pathogenicity is due to motor neuron exhaustion by elevated proteostasis costs, as demonstrated by the increased costs of SOD1 variants in Fig. 6.
2. Second, SOD1 mutants have been found to significantly impair mitochondrial respiration [65][66] and cause metabolic abnormalities[67]. These observations suggest an energetic role of these mutants on ALS pathogenesis, consistent with a mechanism of motor neuron degeneration resulting from energy shortage[68].
3. Third, recently identified genetic risk factors, notably the most important one, the GGGGCC hexanucleotide repeat expansion in an open reading frame on chromosome 9, C9ORF72, accounts for a large fraction of ALS and is most likely associated with abnormal RNA processing and transcriptional inefficiency[14]. Other recently identified genetic risk factors also affect RNA metabolism (TAR-DNA binding protein 43[17][69], FUS[18][19]) or protein processing (SQSTM1[20], VCP[21]). Also, TDP-43, a risk factor in ALS, has been suggested to modulate SOD1 levels[70]. Proteostatic exhaustion as the pathogenic mechanism of neuro-degeneration fits well with these observations acting both on mRNA and protein pools (Fig. 5), since inefficiency in the mRNA turnover would increase costs of maintaining the steady-state mRNA pool, thus increasing the total maintenance costs of the motor neurons.
4. It has also been recently found that SOD1 mRNA levels in SALS are significantly elevated (by ∼30% on average) whereas protein SOD1 levels are roughly similar to controls[71]. This is consistent with transcriptional inefficiency and increased costs of maintaining the mRNA pool. Decreased stability increases costs of SOD1 variant turnover that correlate strongly with disease onset, using unchanged total *A*_i_ (i.e. SOD1 levels), as seen in Fig. 6. This correlation relates to the SOD1-related FALS cases, whereas elevated costs of handling the mRNA pool then relate to some SALS cases, although SALS pathology has also been found to involve misfolded wild-type SOD1 protein forms[72][73].
5. Wild-type SOD1 knock-out mice do not show ALS-like pathology under normal circumstances[4]. Normally this is interpreted as a proof of a gain of toxic function. However, overexpression of wild-type-SOD1 alone does produce motor deficits although to a smaller extent than mutants[74]. These observations are explained by proteostatic exhaustion: Each wild-type copy is less degradation prone than destabilized SOD1 variants but still, high expression increases turnover costs even for the same thermodynamic stability, viz. *A*_i_ in Equation (2). In contrast, knock-out mice cannot express SOD1 and therefore will not be subject to proteostatic exhaustion.
6. There is a critical experiment that can test the validity of the proposed exhaustion model vs. the prevailing “molecular toxicity” model: Expression of SOD1 variants with and without protein turnover. If the proteasome is inhibited in cells expressing SOD1 variants, disease would be aggravated according to the molecular toxicity model, because more toxic misfolded proteins would be available. However, according to the proteome exhaustion model, the disease would be relieved at least to some extent while the cost of protein turnover is reduced to enable normal cell function.
  Importantly, such an experiment was performed in 2014: It was shown that the toxicity of the G85R mutant of SOD1 is small under normal conditions and during inhibition of the proteasome, but when proteasome activity is recovered after washout of the proteasome inhibitor MG132, soluble oligomers of mutant SOD1 correlated strongly with cytotoxicity[75]. This finding is explained by the present exhaustion mechanism: As mutant protein oligomers accumulate, protein degradation becomes increasingly expensive and cause cell death. Proteasome inhibition can lead to several-fold higher levels of SOD1[76]. This would aggravate disease if the protein copies were toxic by themselves, but will reduce the cost of mutant turnover and rescue the cells if proteome exhaustion causes disease. Recently, functionally impaired variants of Ubiquilin-2 have been implicated as a risk factor in ALS[77]. As a central link to the proteasome-mediated degradation of proteins, these findings are consistent with the exhaustion mechanism.
7. Recently, it was shown that treatment of transgenic G37R SOD1 mutant mice with a copper complex increases the steady-state concentration of the expressed mutant SOD1 but also improves motor function and life span[78][79]. It was found that the copper supplement increases the amount of holo-SOD1 due to enhanced metal incorporation. The authors concluded that metal supplements specifically directed towards SOD1 could be a useful therapy. This is consistent with a disease mechanism acting on misfolded proteins (Fig. 6): Rescue of the folded holodimer can then reduce the degradation-targeted misfolded SOD1 pool (U), to improve survival by reducing turnover costs by increasing the amount of folded protein (F).

Some SOD1-mutations reduce metal content to the effect of destabilizing the holoprotein, but in some cases not the apoprotein[40][41]. Since protein misfolding is generally related to metal release[25][39], these mutations, even if they do not destabilize the apoprotein, may be pathogenic for the same reason as the apoprotein-destabilizing mutations: They would increase the pool of non-native apoprotein targeted for degradation, and thus, increase the burden of protein turnover. This may explain why metal-imbalance in the form defined recently (i.e. metal redistribution by loss of functional bound M(II) pool but concomitant enrichment of free chelatable M^2+^ pool [9]) can cause neurological disease, since the turnover of abundant misfolded apoproteins will increase the cellular maintenance energy.

### Concluding remarks

Genotype-phenotype relationships are a major focus area of modern biology, promising improvements in our molecular understanding of disease, diagnostic tools, and personalized therapies. Amyotrophic Lateral Sclerosis (ALS) is an excellent test case of such relationships, with many data available for pathogenic variants of superoxide dismutase 1 (SOD1). In this work, we show the power of such approaches in the quest for disease mechanisms.

Why are neurodegenerative diseases mostly sporadic and have late onset and multiple risk modifiers, including variations in several proteins and non-coding parts of DNA? And yet why do they all associate with protein misfolding, metal dyhomeostasis, metabolic deficiencies, mitochondrial pathologies, and oxidative stress? Why are disease-causing variants spread across the entire protein structure? These facts indirectly point to a systemic impairment of the cells subject to disease. The present work suggests that pathogenesis works strongly via the misfolded protein copies. Both the widely assumed mechanisms of toxic misfolding or aggregation and a general increased energy cost of SOD1 turnover are consistent with these data but only the latter proposed mechanism may also explain several other observations relating to RNA metabolism and bioenergetic effects, as noted above.

When the organism is subject to aging, protein expression levels associated with metabolic, stress, DNA repair and other maintenance controls increase[80]. Since age is the dominant risk factor of late-onset diseases, this aging phenotype needs to be addressed in pathogenic models. The exhaustion mechanism is consistent with this basic fact as exhaustion is likely to become critical when the maintenance costs are increased by aging.

A final question requires answering: Why does ALS occur in the motor neurons, while SOD1 (or its variants) is expressed throughout the body? To see this, one should consider that the motor neurons are among the most energy-requiring cells due to the ATP cost of making action potentials. In a model that emphasizes proteome exhaustion as causing disease, it follows naturally that the cells that have high energy demands will be primarily affected. Importantly, the currently existing molecular toxicity model, which emphasizes molecular modes of toxicity and not energy, does not explain this but would work on any cell type as long as SOD1 is highly expressed. Accordingly, fatigue-resistant motor neurons are less affected than other motor neurons[81].

A very recent computational model by Le Masson et al.[82] shows how vulnerable motor neurons are to energy deficits. While the model does not explain how energy deficits arise, it nicely demonstrates how reduced ATP availability affects action potentials and ion homeostasis, with depolarization as an ultimate consequence. This work this provides a direct link from the exhaustion model to the general homeostasis of the motor neurons.

Since the energy is produced by the mitochondria, with the proteome exhaustion model, it is not surprising that mitochondria accumulate within the neuromuscular junctions, where the SOD1-FALS is thought to begin[83]. This feature of mitochondrial accumulation is directly explained by the exhaustion model and is (also) not explained by the molecular toxicity model.

The proteostatic exhaustion mechanism may apply also to other protein misfolding-related neurodegenerative diseases. These diseases share many commonalities: oxidative stress, aggregated protein deposits, metal ion disorder, diabetes-like pathologies, and metabolic disorders such as impaired glucose utilization[9]. Insoluble aggregates as toxic species are being abandoned[38] in favor of small soluble oligomers in ALS[2][3][4] and Alzheimer’s Disease[9]. According to the present proposed mechanism, this is not due to a toxicity of the oligomers themselves but rather the fact that these abundant, soluble oligomers are (in contrast to aggregates) targeted by the proteasome, causing proteome exhaustion. In contrast, fibrils and aggregates as found in extracellular deposits are non-pathogenic because they do not contribute to the turnover pool, and consequently, one may also infer that inclusion bodies and extracellular aggregates may in fact be ways to protect the cells against the burden of costly protein turnover.

Although changes in SOD1 mRNA levels have been related to SALS, there are only rare and partly conflicting data yet on the transcriptional dysregulation in ALS[84]. However, the genetic risk factors recently identified support a mechanism where energy costs from mRNA turnover aggravate disease[14][17][18][19]. Further studies into these complex mechanisms would help to elucidate the pathogenic mechanisms acting on the mRNA and protein pools, viz. Fig. 5.

## Supporting Information

S1 File Supporting figures and tables. Contains correlations between computed and experimental ΔΔG (Fig. I); collected data for all 150 SOD1 variants (Table I) with references given at the end of the file; computed properties of all 150 variants (Table II); experimental data used for correlation of patient data and stability, charge, and energy cost (Table III); computed copy numbers of unfolded protein and associated relative energy costs (Table IV).
(PDF)
