# Supplementary material for: Genotype-Property Patient-Phenotype Relations Suggest that Proteome Exhaustion Can Cause Amyotrophic Lateral Sclerosis
Source: PLoS One. 2015 Mar 23;10(3):e0118649. doi: 10.1371/journal.pone.0118649 (PMC4370410; doi:10.1371/journal.pone.0118649)
Supplement: S1 File — Contains correlations between computed and experimental ΔΔG (Fig. I); collected data for all 150 SOD1 variants (Table I) with references given at the end of the file; computed properties of all 150 variants (Table II); experimental data used for correlation of patient data and stability, charge, and energy cost (Table III); computed copy numbers of unfolded protein and associated relative energy costs (Table IV). (PDF) [file pone.0118649.s001.pdf]

Supporting Information.

Genotype-Property Patient-Phenotype Relations Suggest that Proteome Exhaustion Causes Amyotrophic Lateral Sclerosis

Kasper P. Kepp*

Department of Chemistry, Technical University of Denmark, DK 2800, Kongens Lyngby, Denmark.

* Corresponding Author (kpj@kemi.dtu.dk). Phone: +045 45 25 24 09.

TABLE S1. 150 ALS-associated SOD1 missense mutations (substitutions) and associated experimentally determined properties: Patient data collected from the ALSOD data base, Wang et al. 2008, with additional references in rightmost column. Data in columns are from left: Site position; type (beta-sheet, B; metal-region, M; disulfide/cystine region, D); wild-type and mutant residue, WT/MUT; free energy change for apomonomer and holodimer in kcal/mol, ΔΔG(m) and ΔΔG(d); number of patient data points, n; time of onset, t(o); survival time, t(s); age of death, t(d) = t(o)+t(s).

SITE	TYPE	WT	MUT	ΔΔG(m)	ΔΔG(d)	n	t(o)	t(s)	t(d)	Reference
3	B	K	E			8	54.2	8.4	63.0	1
4	B	A	S			1	34.0	3.0		2
		A	T			26	45.3	1.5	46.8	3
		A	V		4.31	212	47.7	1.2	48.9	4
		A	F			2	46.0			5
5	B	V	L			1	36	3.8	39.8	6
6	B	C	F			5	50.4	1.0	51.4	7
		C	G			6	49.0	0.2	49.2	8
		C	S			8	50.9	6.7	57.6	9
		C	W			4	67.0	1.9	68.9	7
7	B	V	G							
		V	E			4	48.5	5.7	54.2	10
8	B	L	Q			12	52.3	1.6	53.9	11
		L	V			1	28.0			12
10	B	G	V			5	31.2	1.1	32.3	13
		G	R			1	68.0			14
11	B	D	Y			1	40.0			15
		D	A							16
12	B	G	R			3	60.3	5.5	65.8	17,18
		G	A			1	63.0			
14	B	V	G			2	40.0	1.7	41.7	19
		V	M							20
16	B	G	S			1	18.0			21
		G	A							16
19	B	N	S			1	32.0			22

20	B	F	C			**8**	**51.1**	**2.0**	**53.1**	12
21	B	E	G			**15**	**51.8**	**17.2**	**69.0**	23
		E	K							24
22	B	Q	L							12
		Q	R			1	38.0			25
29	B	V	A							16
31	B	V	A			1	49	>11	**>60**	26
37	B	G	R			**27**	**36.9**	**17.0**	**53.9**	27
		G	V				62.0			28
38	B	L	R			**3**	**42.0**			29
		L	V	**2.25**	**3.24**	**31**	**41.1**	**2.4**	**43.5**	30
41	B	G	D		**3.47**	**17**	**45.2**	**14.1**	**59.3**	31
		G	S		**4.47**	**25**	**48.4**	**1.0**	**49.4**	32
43	B	H	R		**4.05**	**14**	**49.3**	**1.8**	**51.1**	33
45	B	F	C			**3**	**58.0**	**6.0**	**64.0**	34
46	M	H	R		**-0.48**	**70**	**45.6**	**17.6**	**63.2**	35
		H	D							16
47	B	V	F			**1**	**36.0**			36
		V	A							37
48	M	H	R							12
		H	Q			**5**	**48.4**	**1.2**	**49.6**	38
49	B	E	K							29
54	D	T	R							12
57	D	C	R							16
59	D	S	I							12
61	M	G	R							39
64	M	F	L							40
65	M	N	S			**4**	**40.0**			41

66	M	P	S							42
		P	A							16
		P	R							37
67	M	L	P			2	56.0			43
		L	R							29
72	M	G	S			**4**	**37.7**	**3.3**	**41.0**	44
		G	C			**1**	**71.0**	**4.4**		45
76	M	D	V	**0.2**	**0.06**	**5**	**46.0**	**18.8**	**64.8**	46
		D	Y	**0.21**	**0.09**	**3**	**55.0**	**17.0**	**72.0**	19
80	M	H	A							47
		H	R			1	24.0			16
83		D	G							16
84	M	L	F			**26**	**43.3**	**5.8**	**49.1**	48
		L	V		**2.65**	**12**	**46.8**	**3.2**	**50.0**	20
85	M	G	R	**1.20**	**0.90**	**11**	**55.5**	**6.0**	**61.5**	49
		G	S			2	37.5	1.5		50
86	B	N	D	**-0.05**	**0.94**					16
		N	S	**-0.07**	**0.45**	**5**	**40.8**	**6.8**	**47.6**	51
		N	K	**0.66**	**1.41**	**7**	**58.2**	**1.7**	**59.9**	52
		N	I							16
87	B	V	A							12
		V	M							16
89	B	A	T					>3.8		12
		A	V			**5**	**55.6**	**3.8**	**59.4**	53
90	B	D	A	**0.66**	**0.65**	**28**	**48.6**	**8.0**	**56.6**	54
		D	V	**1.41**	**1.85**	**4**	**46.0**	**2.7**	**48.7**	55
		D	N							56
93		G	A		**2.98**	**15**	**46.0**	**3.1**	**49.1**	57
		G	C			**30**	**45.8**	**12.1**	**57.9**	58
		G	D		4.5	**10**	**39.3**	**8.8**	**48.1**	59
		G	R		3	**4**	**35.0**	**5.3**	**40.3**	38
		G	S		3.1	**15**	**51.6**	**8.0**	**59.6**	60
		G	V		5.4	**4**	**43.6**	**6.0**	**49.6**	61
94		V	A							62

95	B	A	G							63
		A	T							41
		A	V							16
96	B	D	N							64
		D	V							
97	B	V	M							12
		V	L							
99	B	I	V			1	60.0			
100	B	E	G	**2.40**	**2.22**	**54**	**47.3**	**4.7**	**52.0**	31
		E	K			1	35.0	17.0		65
101	B	D	G	**0.72**	**1.39**	**2**	**41.0**	**1.9**		66
		D	H			2	52.0	**1.2**		67
		D	N	**-0.80**	**-0.75**	**17**	**40.1**	**2.3**	**42.4**	68
		D	Y			2	46.5	0.9		69
104	B	I	F		**1.24**	**7**	**36.9**	**21.3**	**58.2**	70
105	B	S	L	**1.81**	**2.60**	**8**	**48.7**	**3.5**	**52.2**	71
106	B	L	V		**3.62**	**17**	**45.1**	**1.9**	**47.0**	31
		L	F			**6**	**48.8**	**1.9**	**50.7**	72
108	B	G	V			**5**	**53.6**	**4.0**	**57.6**	73
109	B	D	Y			1	51.0	6.0		
111	B	C	Y							74
112	B	I	M			**28**	**48.5**	**2.2**	**50.7**	27
		I	T			**3**	**45.3**	**0.9**	**46.2**	38,59
113	B	I	F			**3**	**58.7**	**6.0**	**64.7**	75
		I	T		**2.48**	**53**	**54.1**	**6.8**	**60.9**	34
114	B	G	A	**2.33**	**3.27**	**2**	**37.5**	**2.7**		76
115	B	R	G			**3**	**60.0**	**2.5**	**62.5**	77
		R	C			1	74.0	0.9		78
116	B	T	R							
117	B	L	V							

118	B	V	L			1	38.0			12
		V	M							
120		H	L							62
124	M	D	G							12
		D	V			**1**	**42.0**	**>2**		61
125	M	D	H			**5**	**60.2**	**1.8**	**62.0**	38
126	B	L	S			**8**	**52.3**	**8.0**	**60.3**	79
127	B	G	R			1	60.0			80
134	M	S	N			**5**	**53.8**	**1.2**	**55.0**	81
		S	T							82
137	B	T	R			2	42.5			
		T	A			3	60.0			83
138	B	G	E			1	41.0			
139	B	N	H			**9**	**57.9**	**3.0**	**60.9**	84
		N	K	**0.06**	**0.24**			vitro		85
		N	D	**0.01**	**-0.36**					
		N	L							85
140	B	A	G			**5**	**68.2**	**10.0**	**78.2**	86
141	B	G	E			**1**	**43.0**	**3.5**		67
		G	A							62
144	B	L	F		**1.89**	**26**	**52.4**	**11.8**	**64.2**	87
		L	S	**0.2**	**1.07**	**5**	**37.0**	**12.3**	**49.3**	88
145	B	A	G			3	36.7			
		A	T			**2**	**48.0**	**1.6**		88
146	D	C	R			**2**	**55.5**	**2.8**		65
147	B	G	R							12
		G	S			1	64.0			
		G	D							
148	B	V	G		**4.56**	**11**	**43.1**	**2.1**	**45.2**	89
		V	I	**-0.4**		**4**	**28.0**	**1.5**	**29.5**	90

149	B	I	T			**14**	**37.6**	**2.7**	**40.3**	85,91
151	B	I	S							12
		I	T			**2**	**48.5**	**20.0**		

Stabilities (ΔΔG) collected from:

Red numbers: Byström et al., J. Biol. Chem. 2010, 285, 19544.

Blue numbers: Vassall et al., Proc. Natl. Acad. Sci. USA 2011, 108, 2210.

Green numbers: Lindberg et al. Proc. Natl. Acad. Sci. USA 2005, 102, 9754.

Patient data collected from:

Black numbers: References in right column.

Red numbers: Wang et al. PLOS Biol. 2008, 6, e170.

Purple numbers: ALSOD data base. http://alsod.iop.kcl.ac.uk/

Blue numbers: Non-redundant combination of Wang eta l. and ALSOD data base.

Table S2. Computed data for 150 SOD1-mutations in Table S1: SA: Solvent accessibility computed using PoPMuSiC; ΔΔG(POP): stability change from PoPMuSiC2.1; ΔΔG(Imut): stability change from IMutant2.0; dQ: Change in absolute net charge; dHydro: change in hydrophobicity (Rose, G.D. Science 229: 834, 1985); beta: ratio of beta propensities of mutant and WT residues; alpha: ratio of alpha propensities of mutant and WT residues; and corresponding natural logarithms of these ratios.

SITE	WT	MUT	SA	ΔΔG(POP)	ΔΔG(Imut)	dQ	dHydro	beta	alpha	LN(beta)	LN(alpha)
3	K	E	35.9	0.76	0.74	2	-0.14	0.87	1.17	-0.14	0.16
4	A	S	1.7	1.02	1.31	0	-1.63	1.14	0.51	0.14	-0.68
4	A	T	1.7	0.73	1.78	0	-1.39	1.57	0.53	0.45	-0.64
4	A	V	1.7	-0.43	1.08	0	0.91	2.47	0.63	0.91	-0.46
4	A	F	1.7	-0.14	1.16	0	1.88	1.93	0.66	0.66	-0.41
5	V	L	2.1	1.46	0.93	0	0.52	0.61	1.43	-0.49	0.36
6	C	F	1.0	0.15	1.43	0	2.02	1.20	1.32	0.18	0.28
6	C	G	1.0	2.42	3.19	0	-0.25	0.52	0.62	-0.65	-0.48
6	C	S	1.0	2.25	1.85	0	-1.49	0.71	1.01	-0.35	0.01
6	C	W	1.0	0.16	1.49	0	1.88	0.98	1.57	-0.02	0.45
7	V	G	17.6	2.09	3.70	0	-1.30	0.34	0.49	-1.08	-0.70
7	V	E	17.6	1.86	1.83	1	-4.21	0.38	1.48	-0.97	0.39
8	L	Q	0.3	3.36	2.18	0	-3.12	0.69	1.00	-0.38	0.00
8	L	V	0.3	1.33	1.27	0	-0.52	1.63	0.70	0.49	-0.36
10	G	V	33.5	0.01	0.39	0	1.30	2.94	2.02	1.08	0.70
10	G	R	33.5	0.88	0.80	-1	-3.95	1.45	2.61	0.37	0.96
11	D	Y	99.5	0.32	0.07	-1	5.28	2.94	1.06	1.08	0.06
11	D	A	99.5	-0.03	-0.10	-1	4.20	1.52	1.72	0.42	0.54
12	G	R	69.2	0.72	1.04	-1	-3.95	1.45	2.61	0.37	0.96
12	G	A	69.2	0.18	1.33	0	0.39	1.19	3.22	0.17	1.17
14	V	G	3.7	2.81	3.42	0	-1.30	0.34	0.49	-1.08	-0.70
14	V	M	3.7	0.97	1.11	0	-0.34	0.59	1.42	-0.54	0.35
16	G	S	12.5	0.67	0.95	0	-1.24	1.36	1.63	0.31	0.49
16	G	A	12.5	0.34	0.36	0	0.39	1.19	3.22	0.17	1.17
19	N	S	28.4	0.17	0.71	0	0.67	1.36	1.03	0.31	0.03
20	F	C	0.7	2.60	1.41	0	-2.02	0.84	0.76	-0.18	-0.28
21	E	G	25.8	1.73	1.51	-1	2.91	0.90	0.33	-0.10	-1.10
21	E	K	25.8	0.71	2.33	-2	0.14	1.15	0.86	0.14	-0.16
22	Q	L	3.4	-0.15	0.28	0	3.12	1.46	1.00	0.38	0.00
22	Q	R	3.4	0.74	1.76	-1	-2.65	1.18	0.90	0.16	-0.10
29	V	A	0.0	2.57	1.37	0	-0.91	0.40	1.59	-0.91	0.46
31	V	A	0.3	3.54	2.05	0	-0.91	0.40	1.59	-0.91	0.46
37	G	R	46.0	1.65	1.38	-1	-3.95	1.45	2.61	0.37	0.96
37	G	V	46.0	1.85	1.35	0	1.30	2.94	2.02	1.08	0.70
38	L	R	5.2	1.87	2.52	-1	-5.77	0.81	0.90	-0.21	-0.10

38	L	V	5.2	1.35	0.67	0	-0.52	1.63	0.70	0.49	-0.36
41	G	D	26.1	2.49	2.18	1	-3.81	0.78	1.87	-0.25	0.63
41	G	S	26.1	2.47	1.65	0	-1.24	1.36	1.63	0.31	0.49
43	H	R	0.7	1.42	1.33	-1	-3.31	0.94	1.35	-0.06	0.30
45	F	C	0.0	2.69	0.89	0	-2.02	0.84	0.76	-0.18	-0.28
46	H	R	0.0	1.04	0.74	-1	-3.31	0.94	1.35	-0.06	0.30
46	H	D	0.0	2.94	1.21	1	-3.17	0.51	0.97	-0.68	-0.03
47	V	F	0.0	0.65	1.22	0	0.97	0.78	1.05	-0.25	0.05
47	V	A	0.0	2.92	1.19	0	-0.91	0.40	1.59	-0.91	0.46
48	H	R	0.0	1.84	0.74	-1	-3.31	0.94	1.35	-0.06	0.30
48	H	Q	0.0	2.08	0.68	0	-0.66	0.80	1.49	-0.23	0.40
49	E	K	36.8	0.27	0.96	-2	0.14	1.15	0.86	0.14	-0.16
54	T	R	37.1	0.60	0.11	-1	-2.95	0.78	1.54	-0.25	0.43
57	C	R	11.6	2.48	1.11	-1	-4.20	0.76	1.62	-0.28	0.48
59	S	I	18.1	1.02	0.15	0	3.06	1.99	1.41	0.69	0.35
61	G	R	19.9	1.74	0.60	-1	-3.95	1.45	2.61	0.37	0.96
64	F	L	2.7	1.57	2.03	0	-0.45	0.78	1.36	-0.25	0.31
65	N	S	27.5	1.16	0.28	0	0.67	1.36	1.03	0.31	0.03
66	P	S	35.8	-0.15	1.36	0	-2.23	2.18	1.70	0.78	0.53
66	P	A	35.8	-0.08	1.72	0	-0.60	1.90	3.36	0.64	1.21
66	P	R	35.8	0.04	1.14	-1	-4.94	2.33	2.73	0.84	1.00
67	L	P	47.9	1.36	1.01	0	-0.83	0.35	0.33	-1.06	-1.11
67	L	R	47.9	0.18	0.71	-1	-5.77	0.81	0.90	-0.21	-0.10
72	G	S	1.7	0.70	0.57	0	-1.24	1.36	1.63	0.31	0.49
72	G	C	1.7	-0.18	1.32	0	0.25	1.92	1.61	0.65	0.48
76	D	V	35.0	0.43	0.31	-1	5.11	3.76	1.08	1.32	0.08
76	D	Y	35.0	0.70	-0.29	-1	5.28	2.94	1.06	1.08	0.06
80	H	A	6.4	2.86	0.53	0	1.03	0.77	1.66	-0.26	0.51
80	H	R	6.4	1.57	0.48	-1	-3.31	0.94	1.35	-0.06	0.30
83	D	G	0.0	1.49	1.19	-1	3.81	1.28	0.53	0.25	-0.63
84	L	F	1.8	1.14	0.81	0	0.45	1.28	0.74	0.25	-0.31
84	L	V	1.8	1.50	0.75	0	-0.52	1.63	0.70	0.49	-0.36
85	G	R	2.8	2.26	0.70	-1	-3.95	1.45	2.61	0.37	0.96
85	G	S	2.8	2.59	0.65	0	-1.24	1.36	1.63	0.31	0.49
86	N	D	22.7	1.19	1.70	1	-1.90	0.78	1.18	-0.25	0.16
86	N	S	22.7	0.81	0.38	0	0.67	1.36	1.03	0.31	0.03
86	N	K	22.7	0.89	0.87	-1	-0.86	1.28	1.62	0.25	0.48
86	N	I	22.7	-0.08	-0.58	0	3.73	2.70	1.45	0.99	0.37
87	V	A	4.6	2.38	1.82	0	-0.91	0.40	1.59	-0.91	0.46
87	V	M	4.6	1.34	1.13	0	-0.34	0.59	1.42	-0.54	0.35
89	A	T	1.7	0.38	1.66	0	-1.39	1.57	0.53	0.45	-0.64
89	A	V	1.7	-0.44	0.40	0	0.91	2.47	0.63	0.91	-0.46
90	D	A	46.9	0.86	0.58	-1	4.20	0.66	0.58	-0.42	-0.54
90	D	V	46.9	0.72	0.47	-1	5.11	3.76	1.08	1.32	0.08
90	D	N	46.9	0.24	0.22	-1	1.90	1.28	0.85	0.25	-0.16
93	G	A	5.7	2.80	0.48	0	0.39	1.19	3.22	0.17	1.17

93	G	C	5.7	1.56	1.25	0	0.25	1.92	1.61	0.65	0.48
93	G	D	5.7	2.27	1.92	1	-3.81	0.78	1.87	-0.25	0.63
93	G	R	5.7	1.94	1.27	-1	-3.95	1.45	2.61	0.37	0.96
93	G	S	5.7	2.20	1.62	0	-1.24	1.36	1.63	0.31	0.49
93	G	V	5.7	2.78	0.93	0	1.30	2.94	2.02	1.08	0.70
94	V	A	25.8	1.56	0.34	0	-0.91	0.40	1.59	-0.91	0.46
95	A	G	0.0	1.89	2.31	0	-0.39	0.84	0.31	-0.17	-1.17
95	A	T	0.0	1.18	1.75	0	-1.39	1.57	0.53	0.45	-0.64
95	A	V	0.0	0.60	0.42	0	0.91	2.47	0.63	0.91	-0.46
96	D	N	83.5	-0.21	1.02	-1	1.90	1.28	0.85	0.25	-0.16
96	D	V	83.5	-0.61	0.92	-1	5.11	3.76	1.08	1.32	0.08
97	V	M	4.9	1.36	1.32	0	-0.34	0.59	1.42	-0.54	0.35
97	V	L	4.9	1.14	1.27	0	0.52	0.61	1.43	-0.49	0.36
99	I	V	18.0	1.10	-0.08	0	-0.52	1.09	0.88	0.08	-0.13
100	E	G	72.2	0.73	1.26	-1	2.91	0.90	0.33	-0.10	-1.10
100	E	K	72.2	0.02	1.82	-2	0.14	1.15	0.86	0.14	-0.16
101	D	G	6.9	1.53	2.79	-1	3.81	1.28	0.53	0.25	-0.63
101	D	H	6.9	0.54	1.77	-1	3.17	1.98	1.03	0.68	0.03
101	D	N	6.9	0.63	1.86	-1	1.90	1.28	0.85	0.25	-0.16
101	D	Y	6.9	0.12	0.60	-1	5.28	2.94	1.06	1.08	0.06
104	I	F	0.8	0.79	1.01	0	0.45	0.85	0.92	-0.16	-0.08
105	S	L	8.1	1.00	0.98	0	3.06	1.32	1.77	0.28	0.57
106	L	V	6.0	1.00	1.19	0	-0.52	1.63	0.70	0.49	-0.36
106	L	F	6.0	0.69	1.24	0	0.45	1.28	0.74	0.25	-0.31
108	G	V	71.5	1.34	1.32	0	1.30	2.94	2.02	1.08	0.70
109	D	Y	81.9	0.68	0.89	-1	5.28	2.94	1.06	1.08	0.06
111	C	Y	17.5	1.27	0.60	0	1.22	1.20	1.23	0.18	0.21
112	I	M	0.0	1.08	1.08	0	-0.86	0.64	1.25	-0.45	0.22
112	I	T	0.0	1.31	2.34	0	-2.82	0.69	0.74	-0.37	-0.31
113	I	F	11.1	0.93	1.12	0	0.45	0.85	0.92	-0.16	-0.08
113	I	T	11.1	1.53	2.23	0	-2.82	0.69	0.74	-0.37	-0.31
114	G	A	0.0	2.22	0.34	0	0.39	1.19	3.22	0.17	1.17
115	R	G	15.0	1.98	1.59	1	3.95	0.69	0.38	-0.37	-0.96
115	R	C	15.0	0.54	0.75	1	4.20	1.32	0.62	0.28	-0.48
116	T	R	1.0	1.60	1.52	-1	-2.95	0.78	1.54	-0.25	0.43
117	L	V	0.0	1.54	1.12	0	-0.52	1.63	0.70	0.49	-0.36
118	V	L	0.0	1.19	0.15	0	0.52	0.61	1.43	-0.49	0.36
118	V	M	0.0	1.11	0.85	0	-0.34	0.59	1.42	-0.54	0.35
120	H	L	3.2	0.98	-0.60	0	2.46	1.16	1.49	0.15	0.40
124	D	G	3.5	1.10	0.74	-1	3.81	1.28	0.53	0.25	-0.63
124	D	V	3.5	0.32	0.07	-1	5.11	3.76	1.08	1.32	0.08
125	D	H	28.7	0.42	-0.16	-1	3.17	1.98	1.03	0.68	0.03
126	L	S	30.8	0.27	0.71	0	-3.06	0.76	0.56	-0.28	-0.57
127	G	R	11.9	2.13	0.90	-1	-3.95	1.45	2.61	0.37	0.96
134	S	N	0.4	1.51	-0.10	0	-0.67	0.74	0.97	-0.31	-0.03
134	S	T	0.4	1.00	0.22	0	0.24	1.37	1.04	0.31	0.04

137	T	R	41.0	1.15	-0.06	-1	-2.95	0.78	1.54	-0.25	0.43
137	T	A	41.0	0.87	0.22	0	1.39	0.64	1.90	-0.45	0.64
138	G	E	0.0	3.50	1.00	1	-2.91	1.11	3.00	0.10	1.10
139	N	H	30.8	0.73	0.50	0	1.27	1.55	1.22	0.44	0.20
139	N	K	30.8	1.42	0.26	-1	-0.86	1.28	1.62	0.25	0.48
139	N	D	30.8	1.00	0.75	1	-1.90	0.78	1.18	-0.25	0.16
139	N	L	30.8	0.96	-0.93	0	3.73	1.80	1.82	0.59	0.60
140	A	G	0.4	1.51	1.68	0	-0.39	0.84	0.31	-0.17	-1.17
141	G	E	40.9	2.02	0.84	1	-2.91	1.11	3.00	0.10	1.10
141	G	A	40.9	1.76	1.19	0	0.39	1.19	3.22	0.17	1.17
144	L	F	24.1	0.53	0.75	0	0.45	1.28	0.74	0.25	-0.31
144	L	S	24.1	1.20	1.15	0	-3.06	0.76	0.56	-0.28	-0.57
145	A	G	0.0	0.87	2.25	0	-0.39	0.84	0.31	-0.17	-1.17
145	A	T	0.0	0.62	1.04	0	-1.39	0.80	0.53	-0.22	-0.64
146	C	R	3.8	2.54	1.59	-1	-4.20	0.76	1.62	-0.28	0.48
147	G	R	11.9	0.76	1.05	-1	-3.95	1.45	2.61	0.37	0.96
147	G	S	11.9	0.52	0.65	0	-1.24	1.36	1.63	0.31	0.49
147	G	D	11.9	1.34	1.72	1	-3.81	0.78	1.87	-0.25	0.63
148	V	G	13.7	2.09	3.15	0	-1.30	0.34	0.49	-1.08	-0.70
148	V	I	13.7	0.56	0.98	0	0.52	0.92	1.14	-0.08	0.13
149	I	T	0.0	2.60	2.60	0	-2.82	0.69	0.74	-0.37	-0.31
151	I	S	21.6	1.37	1.92	0	-3.06	0.50	0.71	-0.69	-0.35
151	I	T	21.6	0.99	2.10	0	-2.82	0.69	0.74	-0.37	-0.31

Table S3. 30 variants with experimentally known dimer stabilities (kcal/mol) correlated against t(o), t(s), and t(d) (years). Change in net charge is given in the last column. These data are shown in the main paper, Figure 2. Color codes follow literature collections from Table S1.

SITE	TYPE	WT	MUT	ΔΔG(d)	n	t(o)	t(s)	t(d)	ΔQ
4		A	V	4.31	212	47.7	1.2	48.9	0
38		L	V	3.24	31	41.1	2.4	43.5	0
41	B	G	D	3.47	17	45.2	14.1	59.3	-1
41		G	S	4.47	25	48.4	1.0	49.4	0
43	B	H	R	4.05	14	49.3	1.8	51.1	1
46	M	H	R	-0.48	70	45.6	17.6	63.2	1
76	M	D	V	0.06	5	46.0	18.8	64.8	1
76		D	Y	0.09	3	55.0	17	72.0	1
84		L	V	2.65	12	46.8	3.2	50.0	0
85	M	G	R	0.9	11	55.5	6.0	61.5	1
86		N	S	0.45	5	40.8	6.8	47.6	0
86		N	K	1.41	7	58.2	1.7	59.9	1
90	B	D	A	0.65	28	48.6	8.0	56.6	1
90		D	V	1.85	4	46.0	2.7	48.7	1
93		G	A	2.98	15	46.0	3.1	49.1	0
93		G	D	4.5	10	39.3	8.8	48.1	-1
93		G	R	3	4	35.0	5.3	40.3	1
93		G	S	3.1	15	51.6	8.0	59.6	0
93		G	V	5.4	4	43.6	6.0	49.6	0
100	B	E	G	2.22	54	47.3	4.7	52	1
101	B	D	G	1.39	2	41.0	1.9	42.9	1
101		D	N	-0.75	17	40.1	2.3	42.4	1
104	B	I	F	1.24	7	36.9	21.3	58.2	0
105	B	S	L	2.6	8	48.7	3.5	52.2	0
106	B	L	V	3.62	17	45.1	1.9	47	0
113		I	T	2.48	53	54.1	6.8	60.9	0
114	B	G	A	3.27	2	37.5	2.7	40.2	0
144	B	L	F	1.89	26	52.4	11.8	64.2	0
144		L	S	1.07	5	37.0	12.3	49.3	0
148	B	V	G	4.56	11	43.1	2.1	45.2	0

Figure S1. Correlation between ΔΔG computed with POPMUSIC (multiplied by 2 to reflect the dimer estimates) and IMUTANT vs. the experimental holodimer data (all values in kcal/mol) (numerical data in Table S1 (dimer experimental data) and Table S2 (POPMUSIC numbers).

POPMUSIC2.1

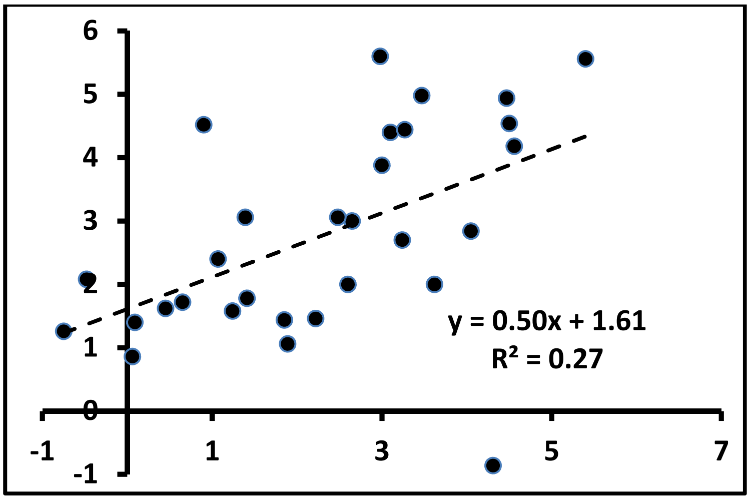

IMUTANT2.0

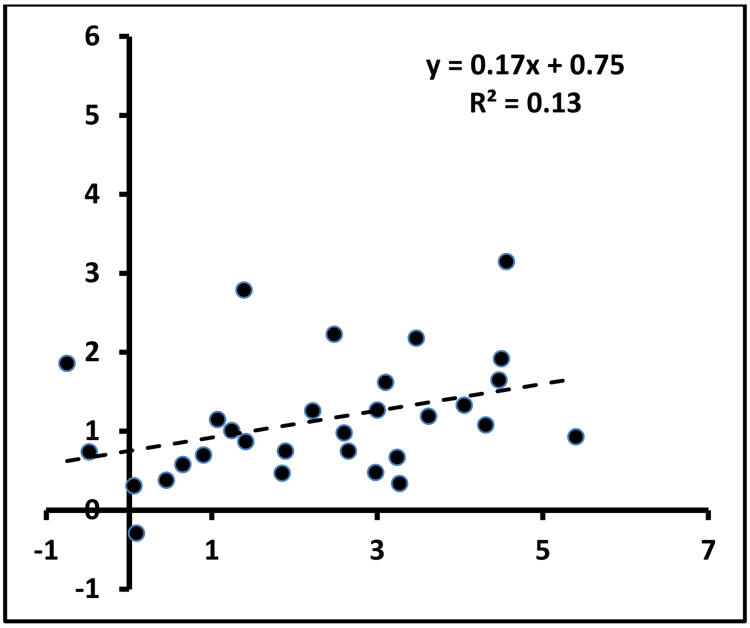

Table S4. Computed steady-state copy numbers from Equation (2) using the experimental ∆Gs, as shown in Figure 6 in the main paper.

SITE	WT	MUT	Ubefore	Uafter	dU	dE_m/dt	t(s)	Ln($\Delta dE_m/dt$)
4	A	V	2.06E-04	2.80E-01	2.80E-01	2.80E-08	1.2	-17.39
38	L	V	2.06E-04	4.67E-02	4.65E-02	4.65E-09	2.4	-19.19
41	G	D	2.06E-04	6.86E-02	6.84E-02	6.84E-09	14.1	-18.80
41	G	S	2.06E-04	3.66E-01	3.65E-01	3.65E-08	1	-17.12
43	H	R	2.06E-04	1.81E-01	1.81E-01	1.81E-08	1.8	-17.83
76	D	V	2.06E-04	2.28E-04	2.18E-05	2.18E-12	18.8	-26.85
76	D	Y	2.06E-04	2.40E-04	3.35E-05	3.35E-12	17	-26.42
84	L	V	2.06E-04	1.74E-02	1.72E-02	1.72E-09	3.2	-20.18
85	G	R	2.06E-04	9.30E-04	7.23E-04	7.23E-11	6	-23.35
86	N	S	2.06E-04	4.38E-04	2.32E-04	2.32E-11	6.8	-24.49
86	N	K	2.06E-04	2.18E-03	1.98E-03	1.98E-10	1.7	-22.34
90	D	A	2.06E-04	6.12E-04	4.06E-04	4.06E-11	8	-23.93
90	D	V	2.06E-04	4.56E-03	4.35E-03	4.35E-10	2.7	-21.56
93	G	A	2.06E-04	3.02E-02	3.00E-02	3.00E-09	3.1	-19.62
93	G	D	2.06E-04	3.84E-01	3.84E-01	3.84E-08	8.8	-17.07
93	G	R	2.06E-04	3.12E-02	3.10E-02	3.10E-09	5.3	-19.59
93	G	S	2.06E-04	3.69E-02	3.67E-02	3.67E-09	8	-19.42
93	G	V	2.06E-04	1.73E+00	1.73E+00	1.73E-07	6	-15.57
100	E	G	2.06E-04	8.47E-03	8.26E-03	8.26E-10	4.7	-20.91
101	D	G	2.06E-04	2.11E-03	1.90E-03	1.90E-10	1.9	-22.38
104	I	F	2.06E-04	1.64E-03	1.44E-03	1.44E-10	21.3	-22.66
105	S	L	2.06E-04	1.60E-02	1.58E-02	1.58E-09	3.5	-20.27

106	L	V	2.06E-04	8.82E-02	8.80E-02	8.80E-09	1.9	-18.55
113	I	T	2.06E-04	1.31E-02	1.29E-02	1.29E-09	6.8	-20.47
114	G	A	2.06E-04	4.91E-02	4.89E-02	4.89E-09	2.7	-19.14
144	L	F	2.06E-04	4.87E-03	4.67E-03	4.67E-10	11.8	-21.49
144	L	S	2.06E-04	1.24E-03	1.03E-03	1.03E-10	12.3	-23.00
148	V	G	2.06E-04	4.25E-01	4.25E-01	4.25E-08	2.1	-16.97

References

[1] Kúzma-Kozakiewicz, M.; Berdynski, M.; Morita, M.; takahashi, Y.; kawata, A. et al. Amyotr. Lat. Scleros. Frontotemp. Deg. 2013, 14, 608-614.

[2] Wang, Q.; Johnson, J. L.; Agar, N. Y.; Agar, J. N. PLOS Biol. 2008, 6, e170.

[3] Nakano R, Sato S, Inuzuka T, Sakimura K, Mishina M, et al. A novel mutation in Cu/Zn superoxide dismutase gene in Japanese familial amyotrophic lateral sclerosis. Biochem Biophys Res Commun. 1994, 200, 695-703.

[4] Deng HX, Hentati A, Tainer JA, et al. Amyotrophic lateral sclerosis and structural defects in Cu,Zn superoxide dismutase. Science 1993;261:1047–1051.

[5] Baek W, Koh SH, Park JS, Kim YS, Kim HY, Kwon MJ, Ki CS, Kim SH. A novel codon4 mutation (A4F) in the SOD1gene in familial amyotrophic lateral sclerosis. J Neurol Sci. 2011;306(1-2):157-159

[6] Weber M, Neuwirth C, Thierbach J, Schweikert K, Czaplinski A, Petersen J, Jung HH, Birve A, Marklund SL, Andersen PM. ALS patients with SOD1 mutations in Switzerland show very diverse phenotypes and extremely long survival. J Neurol Neurosurg Psychiatry. 2012;83(3):351-353.

[7] Wang Z, Cai W, Cui F, Cai T, Chen Z, Mao F, Teng H, Chen L, Wang J, Sun Z, Huang X, Yu P. Identification of a novel missense (C7W) mutation of SOD1 in a large familial amyotrophic lateral sclerosis pedigree. Neurobiol Aging. 2014 35(3):725.e11-5.

[8] Kohno S, Takahashi Y, Miyajima H, Serizawa M, Mizoguchi K. A novel mutation (Cys6Gly) in the Cu/Zn superoxide dismutase gene associated with rapidly progressive familial amyotrophic lateral sclerosis. Neurosci Lett. 1999;276(2):135-137.

[9] Brotherton T, Polak M, Kelly C, Birve A, Andersen P, Marklund SL, Glass JD. A novel ALS SOD1 C6S mutation with implications for aggregation related toxicity and genetic counseling. Amyotroph Lateral Scler. 2011;12(3):215-219.

[10] Hirano M, Fujii J, Nagai Y, Sonobe M, Okamoto K, Araki H, Taniguchi N, Ueno S. A new variant Cu/Zn superoxide dismutase (Val7-->Glu) deduced from lymphocyte mRNA sequences from Japanese patients with familial amyotrophic lateral sclerosis. Biochem Biophys Res Commun. 1994, 28;204(2):572-577.

[11] Bereznai B, Winkler A, Borasio GD, Gasser T. A novel SOD1 mutation in an Austrian family with amyotrophic lateral sclerosis. Neuromuscul Disord. 1997;7(2):113-116.

[12] Andersen PM, Sims KB, Xin WW, Kiely R, O'Neill G, Ravits J, Pioro E, Harati Y, Brower RD, Levine JS, Heinicke HU, Seltzer W, Boss M, Brown RH Jr. Sixteen novel mutations in the Cu/Zn superoxide dismutase gene in amyotrophic lateral sclerosis: a decade of discoveries, defects and disputes. Amyotroph Lateral Scler Other Motor Neuron Disord. 2003;4(2):62-73.

[13] Kim NH, Kim HJ, Kim M, Lee KW. A novel SOD1 gene mutation in a Korean family with amyotrophic lateral sclerosis. J Neurol Sci. 2003;206(1):65-9.

[14] Ricci C, Benigni M, Battistini S, Greco G, Torzini A, Giannini F. A novel exon 1 mutation (G10R) in the SOD1 gene in a patient with familial ALS. Amyotroph Lateral Scler. 2010, 11(5):481-485.

[15] Georgoulopoulou E1, Gellera C, Bragato C, Sola P, Chiari A, Bernabei C, Mandrioli J. A novel SOD1 mutation in a young amyotrophic lateral sclerosis patient with a very slowly progressive clinical course. Muscle Nerve. 2010;42(4):596-597.

[16] Abel O, Powell JF, Andersen PM, Al-Chalabi A.Hum Mutat. 2012 33(9):1345-51. ALSoD: A user-friendly online bioinformatics tool for amyotrophic lateral sclerosis genetics. http://alsod.iop.kcl.ac.uk/

[17] Penco S, Schenone A, Bordo D, Bolognesi M, Abbruzzese M, Bugiani O, Ajmar F, Garrè C. A SOD1 gene mutation in a patient with slowly progressing familial ALS. Neurology. 1999, 53(2):404-406.

[18] Battistini S, Giannini F, Greco G, Bibbò G, Ferrera L, Marini V, Causarano R, Casula M, Lando G, Patrosso MC, Caponnetto C, Origone P, Marocchi A, Del Corona A, Siciliano G, Carrera P, Mascia V, Giagheddu M, Carcassi C, Orrù S, Garrè C, Penco S. SOD1 mutations in amyotrophic lateral sclerosis. Results from a multicenter Italian study. J Neurol. 2005;252(7):782-788.

[19] Andersen PM, Nilsson P, Keränen ML, Forsgren L, Hägglund J, Karlsborg M, Ronnevi LO, Gredal O, Marklund SL. Phenotypic heterogeneity in motor neuron disease patients with CuZn-superoxide dismutase mutations in Scandinavia. Brain. 1997;120 (Pt 10):1723-1737.

[20] Deng HX, Tainer JA, Mitsumoto H, Ohnishi A, He X, Hung WY, Zhao Y, Juneja T, Hentati A, Siddique T. Two novel SOD1 mutations in patients with familial amyotrophic lateral sclerosis. Hum Mol Genet. 1995;4(6):1113-1116.

[21] Kawamata J, Shimohama S, Takano S, Harada K, Ueda K, Kimura J. Novel G16S (GGC-AGC) mutation in the SOD-1 gene in a patient with apparently sporadic young-onset amyotrophic lateral sclerosis. Hum Mutat. 1997;9(4):356-358.

[22] Mayeux V, Corcia P, Besson G, Jafari-Schluep HF, Briolotti V, Camu W. N19S, a new SOD1 mutation in sporadic amyotrophic lateral sclerosis: no evidence for disease causation. Ann Neurol. 2003 53(6):815-818.

[23] Syriani E, Morales M, Gamez J. The p.E22G mutation in the Cu/Zn superoxide-dismutase gene predicts a long survival time: clinical and genetic characterization of a seven-generation ALS1 Spanish pedigree. J Neurol Sci. 2009;285(1-2):46-53.

[24] Jones CT, Swingler RJ, Brock DJ. Identification of a novel SOD1 mutation in an apparently sporadic amyotrophic lateral sclerosis patient and the detection of Ile113Thr in three others. Hum Mol Genet. 1994;3(4):649-650.

[25] Corti S, Donadoni C, Ronchi D, Bordoni A, Fortunato F, Santoro D, Del Bo R, Lucchini V, Crugnola V, Papadimitriou D, Salani S, Moggio M, Bresolin N, Comi GP. Amyotrophic lateral sclerosis linked to a novel SOD1 mutation with muscle mitochondrial dysfunction. J Neurol Sci. 2009;276(1-2):170-174.

[26] Dangoumau A, Verschueren A, Hammouche E, Papon MA, Blasco H, Cherpi-Antar C, Pouget J, Corcia P, Andres CR, Vourch P. A novel SOD1 mutation p.V31A identified with a slowly progressive form of amyotrophic lateral sclerosis. Neurobiol Aging. 2014;35(1):266.e1-4.

[27] García-Redondo A, Bustos F, Juan Y Seva B, Del Hoyo P, Jiménez S, Campos Y, Martín MA, Rubio JC, Cañadillas F, Arenas J, Esteban J. Molecular analysis of the superoxide dismutase 1 gene in Spanish patients with sporadic or familial amyotrophic lateral sclerosis. Muscle Nerve. 2002;26(2):274-278.

[28] Kobayashi J, Kuroda M, Kawata A, Mochizuki Y, Mizutani T, Komori T, Ikeuchi T, Koide R. Novel G37V mutation of SOD1 gene in autopsied patient with familial amyotrophic lateral sclerosis. Amyotroph Lateral Scler. 2012;13(6):570-572.

[29] Boukaftane Y, Khoris J, Moulard B, Salachas F, Meininger V, Malafosse A, Camu W, Rouleau GA. Identification of six novel SOD1 gene mutations in familial amyotrophic lateral sclerosis. Can J Neurol Sci. 1998;25(3):192-196.

[30] Aguirre T, Matthijs G, Robberecht W, Tilkin P, Cassiman JJ. Mutational analysis of the Cu/Zn superoxide dismutase gene in 23 familial and 69 sporadic cases of amyotrophic lateral sclerosis in Belgium. Eur J Hum Genet. 1999;7(5):599-602.

[31] Rosen DR, Siddique T, Patterson D, Figlewicz DA, Sapp P, Hentati A, Donaldson D, Goto J, O'Regan JP, Deng HX, et al. Mutations in Cu/Zn superoxide dismutase gene are associated with familial amyotrophic lateral sclerosis. Nature. 1993;362:59-62.

[32] Rainero I, Pinessi L, Tsuda T, Vignocchi MG, Vaula G, Calvi L, Cerrato P, Rossi B, Bergamini L, McLachlan DR, et al. SOD1 missense mutation in an Italian family with ALS. Neurology. 1994;44(2):347-349.

[33] DiDonato M, Craig L, Huff ME, Thayer MM, Cardoso RM, Kassmann CJ, Lo TP, Bruns CK, Powers ET, Kelly JW, Getzoff ED, Tainer JA. ALS mutants of human superoxide dismutase form fibrous aggregates via framework destabilization. J Mol Biol. 2003;332(3):601-615.

[34] Gellera C, Castellotti B, Riggio MC, Silani V, Morandi L, Testa D, Casali C, Taroni F, Di Donato S, Zeviani M, Mariotti C. Superoxide dismutase gene mutations in Italian patients with familial and sporadic amyotrophic lateral sclerosis: identification of three novel missense mutations. Neuromuscul Disord. 2001;11(4):404-410.

[35] Aoki M, Ogasawara M, Matsubara Y, Narisawa K, Nakamura S, Itoyama Y, Abe K. Familial amyotrophic lateral sclerosis (ALS) in Japan associated with H46R mutation in Cu/Zn superoxide dismutase gene: a possible new subtype of familial ALS. J Neurol Sci. 1994;126(1):77-83.

[36] Gellera C. Genetics of ALS in Italian families. Amyotroph Lateral Scler Other Motor Neuron Disord. 2001;2 Suppl 1:S43-S46.

[37] Millecamps S, Salachas F, Cazeneuve C, Gordon P, Bricka B, Camuzat A, Guillot-Noël L, Russaouen O, Bruneteau G, Pradat PF, Le Forestier N, Vandenberghe N, Danel-Brunaud V, Guy N, Thauvin-Robinet C, Lacomblez L, Couratier P, Hannequin D, Seilhean D, Le Ber I, Corcia P, Camu W, Brice A, Rouleau G, LeGuern E, Meininger V. SOD1, ANG, VAPB, TARDBP, and FUS mutations in familial amyotrophic lateral sclerosis: genotype-phenotype correlations. J Med Genet. 2010;47(8):554-560.

[38] Enayat ZE, Orrell RW, Claus A, Ludolph A, Bachus R, Brockmüller J, Ray-Chaudhuri K, Radunovic A, Shaw C, Wilkinson J, et al. Two novel mutations in the gene for copper zinc superoxide dismutase in UK families with amyotrophic lateral sclerosis. Hum Mol Genet. 1995;4(7):1239-12340.

[39] Conforti FL, Barone R, Fermo SL, Giliberto C, Patti F, Gambardella A, Quattrone A, Zappia M. Sporadic motor neuron disease in a familial novel SOD1 mutation: incomplete penetrance or chance association? Amyotroph Lateral Scler. 2011;12(3):220-222.

[40] Klein, C. J. et al. J. Neurolo. Neurosurg. Psychiatry 2013, 84, 943-944.

[41] Corrado L, D'Alfonso S, Bergamaschi L, Testa L, Leone M, Nasuelli N, Momigliano-Richiardi P, Mazzini L. SOD1 gene mutations in Italian patients with Sporadic Amyotrophic Lateral Sclerosis (ALS). Neuromuscul Disord. 2006;16(11):800-804.

[42] Keckarević D1, Stević Z, Keckarević-Marković M, Kecmanović M, Romac S. A novel P66S mutation in exon 3 of the SOD1 gene with early onset and rapid progression. Amyotroph Lateral Scler. 2012;13(2):237-240.

[43] del Grande A, Luigetti M, Conte A, Mancuso I, Lattante S, Marangi G, Stipa G, Zollino M, Sabatelli M. A novel L67P SOD1 mutation in an Italian ALS patient. Amyotroph Lateral Scler. 2011;12(2):150-2.

[44] Shaw CE, Enayat ZE, Chioza BA, Al-Chalabi A, Radunovic A, Powell JF, Leigh PN. Mutations in all five exons of SOD-1 may cause ALS. Ann Neurol. 1998;43(3):390-394.

[45] Stewart HG, Mackenzie IR, Eisen A, Brännström T, Marklund SL, Andersen PM. Clinicopathological phenotype of ALS with a novel G72C SOD1 gene mutation mimicking a myopathy. Muscle Nerve. 2006;33(5):701-706.

[46] Segovia-Silvestre T1, Andreu AL, Vives-Bauza C, Garcia-Arumi E, Cervera C, Gamez J. A novel exon 3 mutation (D76V) in the SOD1 gene associated with slowly progressive ALS. Amyotroph Lateral Scler Other Motor Neuron Disord. 2002, 3(2):69-74.

[47] Alexander MD, Traynor BJ, Miller N, Corr B, Frost E, McQuaid S, Brett FM, Green A, Hardiman O. "True" sporadic ALS associated with a novel SOD-1 mutation. Ann Neurol. 2002;52(5):680-683.

[48] Ceroni M, Malaspina A, Poloni TE, Alimonti D, Rognoni F, Habgood J, Imbesi F, Antonelli P, Alfonsi E, Curti D, deBelleroche J. Clustering of ALS patients in central Italy due to the occurrence of the L84F SOD1 gene mutation. Neurology. 1999;53(5):1064-1071.

[49] Cao X, Antonyuk SV, Seetharaman SV, Whitson LJ, Taylor AB, Holloway SP, Strange RW, Doucette PA, Valentine JS, Tiwari A, Hayward LJ, Padua S, Cohlberg JA, Hasnain SS, Hart PJ. Structures of the G85R variant of SOD1 in familial amyotrophic lateral sclerosis. J Biol Chem. 2008;283(23):16169-16177.

[50] Takazawa T, Ikeda K, Hirayama T, Kawabe K, Nakamura Y, Ito H, Kano O, Yoshii Y, Tanaka F, Sobue G, Iwasaki Y. Familial amyotrophic lateral sclerosis with a novel G85S mutation of superoxide dismutase 1 gene: clinical features of lower motor neuron disease. Intern Med. 2010;49(2):183-186.

[51] Hayward C, Brock DJ, Minns RA, Swingler RJ. Homozygosity for Asn86Ser mutation in the CuZn-superoxide dismutase gene produces a severe clinical phenotype in a juvenile onset case of familial amyotrophic lateral sclerosis. J Med Genet. 1998;35(2):174.

[52] Beck M, Sendtner M, Toyka KV. Novel SOD1 N86K mutation is associated with a severe phenotype in familial ALS. Muscle Nerve. 2007;36(1):111-114.

[53] Rezania K, Yan J, Dellefave L, Deng HX, Siddique N, Pascuzzi RT, Siddique T, Roos RP. A rare Cu/Zn superoxide dismutase mutation causing familial amyotrophic lateral sclerosis with variable age of onset, incomplete penetrance and a sensory neuropathy. Amyotroph Lateral Scler Other Motor Neuron Disord. 2003;4(3):162-166.

[54] Andersen PM, Forsgren L, Binzer M, Nilsson P, Ala-Hurula V, Keränen ML, Bergmark L, Saarinen A, Haltia T, Tarvainen I, Kinnunen E, Udd B, Marklund SL. Autosomal recessive adult-onset amyotrophic lateral sclerosis associated with homozygosity for Asp90Ala CuZn-superoxide dismutase mutation. A clinical and genealogical study of 36 patients. Brain 1996;119 (Pt 4):1153-1172.

[55] Morita M, Abe K, Takahashi M, Onodera Y, Okomura H, et al. Eur. J. Neurol. 2003, 5, 389-392.

[56] Calvo A, Ilardi A, Moglia C, Canosa A, Carrara G, Valentini C, Ossola I, Brunetti M, Restagno G, Chiò A. An ALS case with a novel D90N-SOD1 heterozygous missense mutation. Amyotroph Lateral Scler. 2012, 13(4):393-395.

[57] Banci L, Bertini I, Boca M, Girotto S, Martinelli M, Valentine JS, Vieru M. SOD1 and amyotrophic lateral sclerosis: mutations and oligomerization. PLoS One. 2008;3(2):e1677.

[58] Régal L, Vanopdenbosch L, Tilkin P, Van den Bosch L, Thijs V, Sciot R, Robberecht W. The G93C mutation in superoxide dismutase 1: clinicopathologic phenotype and prognosis. Arch Neurol. 2006;63(2):262-267.

[59] Esteban J, Rosen DR, Bowling AC, Sapp P, McKenna-Yasek D, O'Regan JP, Beal MF, Horvitz HR, Brown RH Jr. Identification of two novel mutations and a new polymorphism in the gene for Cu/Zn superoxide dismutase in patients with amyotrophic lateral sclerosis. Hum Mol Genet. 1994;3(6):997-998.

[60] Kawata A, Kato S, Hayashi H, Hirai S. Prominent sensory and autonomic disturbances in familial amyotrophic lateral sclerosis with a Gly93Ser mutation in the SOD1 gene. J Neurol Sci. 1997;153(1):82-85.

[61] Hosler BA, Nicholson GA, Sapp PC, Chin W, Orrell RW, de Belleroche JS, Esteban J, Hayward LJ, Mckenna-Yasek D, Yeung L, Cherryson AK, Dench JE, Wilton SD, Laing NG, Horvitz HR, Brown RH Jr. Three novel mutations and two variants in the gene for Cu/Zn superoxide dismutase in familial amyotrophic lateral sclerosis. Neuromuscul Disord. 1996;6(5):361-6.

[62] Brown JA, Min J, Staropoli JF, Collin E, Bi S, Feng X, Barone R, Cao Y, O'Malley L, Xin W, Mullen TE, Sims KB. SOD1, ANG, TARDBP and FUS mutations in amyotrophic lateral sclerosis: a United States clinical testing lab experience. Amyotroph Lateral Scler. 2012, 13(2):217-222.

[63] Chiò A, Borghero G, Pugliatti M, Ticca A, Calvo A, Moglia C, Mutani R, Brunetti M, Ossola I, Marrosu MG, Murru MR, Floris G, Cannas A, Parish LD, Cossu P, Abramzon Y, Johnson JO, Nalls MA, Arepalli S, Chong S, Hernandez DG, Traynor BJ, Restagno G. Large proportion of amyotrophic lateral sclerosis cases in Sardinia due to a single founder mutation of the TARDBP gene. Arch Neurol. 2011;68(5):594-598.

[64] Hand CK, Mayeux-Portas V, Khoris J, Briolotti V, Clavelou P, Camu W, Rouleau GA. Compound heterozygous D90A and D96N SOD1 mutations in a recessive amyotrophic lateral sclerosis family. Ann Neurol. 2001;49(2):267-71.

[65] Siddique T, Deng HX. Genetics of amyotrophic lateral sclerosis. Hum Mol Genet. 1996;5 Spec No:1465-1470.

[66] Yulug IG, Katsanis N, de Belleroche J, Collinge J, Fisher EM. An improved protocol for the analysis of SOD1 gene mutations, and a new mutation in exon 4. Hum Mol Genet. 1995;4(6):1101-1104.

[67] Sato T, Yamamoto Y, Nakanishi T, Fukada K, Sugai F, Zhou Z, Okuno T, Nagano S, Hirata S, Shimizu A, Sakoda S. Identification of two novel mutations in the Cu/Zn superoxide dismutase gene with familial amyotrophic lateral sclerosis: mass spectrometric and genomic analyses. J Neurol Sci. 2004;218(1-2):79-83.

[68] Jones CT, Shaw PJ, Chari G, Brock DJ. Identification of a novel exon 4 SOD1 mutation in a sporadic amyotrophic lateral sclerosis patient. Mol Cell Probes. 1994;8(4):329-330.

[69] Tan CF, Piao YS, Hayashi S, Obata H, Umeda Y, Sato M, Fukushima T, Nakano R, Tsuji S, Takahashi H. Familial amyotrophic lateral sclerosis with bulbar onset and a novel Asp101Tyr Cu/Zn superoxide dismutase gene mutation. Acta Neuropathol. 2004;108(4):332-336.

[70] Ikeda M, Abe K, Aoki M, Sahara M, Watanabe M, Shoji M, St George-Hyslop PH, Hirai S, Itoyama Y. Variable clinical symptoms in familial amyotrophic lateral sclerosis with a novel point mutation in the Cu/Zn superoxide dismutase gene.
Neurology. 1995;45(11):2038-2042.

[71] Zhang H, Zhao H, Lu M, Zhang Y, Wang L, Zhang J, Ma D, Fan D. A rare Cu/Zn superoxide dismutase mutation causing familial amyotrophic lateral sclerosis with variable age of onset and incomplete penetrance in China. Amyotroph Lateral Scler Other Motor Neuron Disord. 2005;6(4):234-238.
[72] Battistini S, Ricci C, Lotti EM, Benigni M, Gagliardi S, Zucco R, Bondavalli M, Marcello N, Ceroni M, Cereda C. Severe familial ALS with a novel exon 4 mutation (L106F) in the SOD1 gene. J Neurol Sci. 2010;293(1-2):112-5.
[73] Orrell RW, Habgood JJ, Malaspina A, Mitchell J, Greenwood J, Lane RJ, deBelleroche JS. Clinical characteristics of SOD1 gene mutations in UK families with ALS. J Neurol Sci. 1999;169(1-2):56-60.
[74] Eisen A, Mezei MM, Stewart HG, Fabros M, Gibson G, Andersen PM. SOD1 gene mutations in ALS patients from British Columbia, Canada: clinical features, neurophysiology and ethical issues in management. Amyotroph Lateral Scler. 2008 Apr;9(2):108-119.
[75] Hermann A, Reuner U, Ziethe G, Bräuer A, Gölnitz U, Rolfs A, Ricci C. Vocal cord paralysis and rapid progressive motor neuron disease by the I113F mutation in SOD1 gene. Amyotroph Lateral Scler. 2011;12(5):382-384.
[76] Jacobsson J1, Jonsson PA, Andersen PM, Forsgren L, Marklund SL. Superoxide dismutase in CSF from amyotrophic lateral sclerosis patients with and without CuZn-superoxide dismutase mutations. Brain. 2001;124(Pt 7):1461-1466.
[77] Kostrzewa M, Burck-Lehmann U, Müller U. Autosomal dominant amyotrophic lateral sclerosis: a novel mutation in the Cu/Zn superoxide dismutase-1 gene. Hum Mol Genet. 1994;3(12):2261-2262.
[78] Tortelli R, Conforti FL, Cortese R, D'Errico E, Distaso E, Mazzei R, Ungaro C, Magariello A, Gambardella A, Logroscino G, Simone IL. Amyotrophic lateral sclerosis: a new missense mutation in the SOD1 gene. Neurobiol Aging. 2013, 34(6):1709. e3-5.
[79] Takehisa Y, Ujike H, Ishizu H, Terada S, Haraguchi T, Tanaka Y, Nishinaka T, Nobukuni K, Ihara Y, Namba R, Yasuda T, Nishibori M, Hayabara T, Kuroda S. Familial amyotrophic lateral sclerosis with a novel Leu126Ser mutation in the copper/zinc superoxide dismutase gene showing mild clinical features and lewy body-like hyaline inclusions. Arch Neurol. 2001;58(5):736-740.
[80] Holmøy T, Wilson JA, von der Lippe C, Andersen PM, Berg-Hansen P. G127R: A novel SOD1 mutation associated with rapidly evolving ALS and severe pain syndrome. Amyotroph Lateral Scler. 2010;11(5):478-480.
[81] Watanabe M, Aoki M, Abe K, Shoji M, Iizuka T, Ikeda Y, Hirai S, Kurokawa K, Kato T, Sasaki H, Itoyama Y. A novel missense point mutation (S134N) of the Cu/Zn superoxide dismutase gene in a patient with familial motor neuron disease. Hum Mutat. 1997;9(1):69-71.

[82] Cui F, Cai W, Wang Z, Ren Y, Li M, Sun Z, Huang X. New mutation in the SOD1 (copper/zinc superoxide dismutase-1) gene in a Chinese amyotrophic lateral sclerosis (ALS) patient. Amyotroph Lateral Scler Frontotemporal Degener. 2013, 14(7-8): 635-637.

[83] Visani M, de Biase D, Bartolomei I, Plasmati R, Morandi L, Cenacchi G, Salvi F, Pession A. A novel T137A SOD1 mutation in an Italian family with two subjects affected by amyotrophic lateral sclerosis. Amyotroph Lateral Scler. 2011, 12(5):385-388.

[84] Nogales-Gadea G, Garcia-Arumi E, Andreu AL, Cervera C, Gamez J. A novel exon 5 mutation (N139H) in the SOD1 gene in a Spanish family associated with incomplete penetrance. J Neurol Sci. 2004;219(1-2):1-6.

[85] Pramatarova A, Figlewicz DA, Krizus A, Han FY, Ceballos-Picot I, Nicole A, Dib M, Meininger V, Brown RH, Rouleau GA. Identification of new mutations in the Cu/Zn superoxide dismutase gene of patients with familial amyotrophic lateral sclerosis. Am J Hum Genet. 1995;56(3):592-596.

[86] Naini A, Musumeci O, Hayes L, Pallotti F, Del Bene M, Mitsumoto H. Identification of a novel mutation in Cu/Zn superoxide dismutase gene associated with familial amyotrophic lateral sclerosis. J Neurol Sci. 2002;198(1-2):17-19.

[87] Masè G, Ros S, Gemma A, Bonfigli L, Carraro N, Cazzato G, Rolfo M, Zanconati F, Sepcic J, Jurjevic A, Pirulli D, Boniotto M, Zezlina S, Crovella S, Amoroso A. ALS with variable phenotypes in a six-generation family caused by leu144phe mutation in the SOD1 gene. J Neurol Sci. 2001, 191(1-2):11-18.

[88] Sapp PC, Rosen DR, Hosler BA, Esteban J, McKenna-Yasek D, O'Regan JP, Horvitz HR, Brown RH Jr. Identification of three novel mutations in the gene for Cu/Zn superoxide dismutase in patients with familial amyotrophic lateral sclerosis. Neuromuscul Disord. 1995;5(5):353-357.

[89] Ghadge GD, Lee JP, Bindokas VP, Jordan J, Ma L, Miller RJ, Roos RP. Mutant superoxide dismutase-1-linked familial amyotrophic lateral sclerosis: molecular mechanisms of neuronal death and protection. J Neurosci. 1997;17(22):8756-8766.

[90] Ikeda M, Abe K, Aoki M, Ogasawara M, Kameya T, Watanabe M, Shoji M, Hirai S, Itoyama Y. A novel point mutation in the Cu/Zn superoxide dismutase gene in a patient with familial amyotrophic lateral sclerosis. Hum Mol Genet. 1995;4(3):491-492.

[91] Fong GC, Kwok KH, Song YQ, Cheng TS, Ho PW, Chu AC, Kung MH, Chan KH, Mak W, Cheung RT, Ramsden DB, Ho SL. Clinical phenotypes of a large Chinese multigenerational kindred with autosomal dominant familial ALS due to Ile149Thr SOD1 gene mutation. Amyotroph Lateral Scler. 2006;7(3):142-149.
